# A unified Foot and Mouth Disease dataset for Uganda: evaluating machine learning predictive performance degradation under varying distributions

**DOI:** 10.3389/frai.2024.1446368

**Published:** 2024-07-31

**Authors:** Geofrey Kapalaga, Florence N. Kivunike, Susan Kerfua, Daudi Jjingo, Savino Biryomumaisho, Justus Rutaisire, Paul Ssajjakambwe, Swidiq Mugerwa, Yusuf Kiwala

**Affiliations:** ^1^Department of Information Technology, College of Computing and Information Sciences, Makerere University, Kampala, Uganda; ^2^National Livestock Resources Research Institute, Kampala, Uganda; ^3^African Center of Excellence in Bioinformatics (ACE-B), Makerere University, Kampala, Uganda; ^4^Department of Computer Science, College of Computing and Information Sciences, Makerere University, Kampala, Uganda; ^5^College of Veterinary Medicine, Animal Resources and Bio-Security, Makerere University, Kampala, Uganda; ^6^College of Business and Management Sciences, Makerere University, Kampala, Uganda

**Keywords:** Foot and Mouth Disease, machine learning, distribution shifts, performance degradation rates, class imbalance

## Abstract

In Uganda, the absence of a unified dataset for constructing machine learning models to predict Foot and Mouth Disease outbreaks hinders preparedness. Although machine learning models exhibit excellent predictive performance for Foot and Mouth Disease outbreaks under stationary conditions, they are susceptible to performance degradation in non-stationary environments. Rainfall and temperature are key factors influencing these outbreaks, and their variability due to climate change can significantly impact predictive performance. This study created a unified Foot and Mouth Disease dataset by integrating disparate sources and pre-processing data using mean imputation, duplicate removal, visualization, and merging techniques. To evaluate performance degradation, seven machine learning models were trained and assessed using metrics including accuracy, area under the receiver operating characteristic curve, recall, precision and F1-score. The dataset showed a significant class imbalance with more non-outbreaks than outbreaks, requiring data augmentation methods. Variability in rainfall and temperature impacted predictive performance, causing notable degradation. Random Forest with borderline SMOTE was the top-performing model in a stationary environment, achieving 92% accuracy, 0.97 area under the receiver operating characteristic curve, 0.94 recall, 0.90 precision, and 0.92 F1-score. However, under varying distributions, all models exhibited significant performance degradation, with random forest accuracy dropping to 46%, area under the receiver operating characteristic curve to 0.58, recall to 0.03, precision to 0.24, and F1-score to 0.06. This study underscores the creation of a unified Foot and Mouth Disease dataset for Uganda and reveals significant performance degradation in seven machine learning models under varying distributions. These findings highlight the need for new methods to address the impact of distribution variability on predictive performance.

## 1 Introduction

*Foot and Mouth Disease* (FMD) is a highly contagious disease primarily affecting cloven-hoofed animals such as cattle, pigs, sheep, and goats (Udahemuka et al., 2020; Chepkwony et al., 2021). FMD is caused by an aphthovirus of the family Picornaviridae, inducing fever and blister-like sores in the mouth and feet of susceptible animals (Childs et al., 2022). While adult animals usually survive, morbidity rates can reach 100% in susceptible populations, especially among young livestock (Bertram et al., 2020; Rodríguez-Habibe et al., 2020). Clinical symptoms include vesicles or blisters on the tongue, hooves, mouth, and udder, leading to lameness and reduced appetite (Alexandersen et al., 2019; Clemmons et al., 2021).

**Figure 1:**
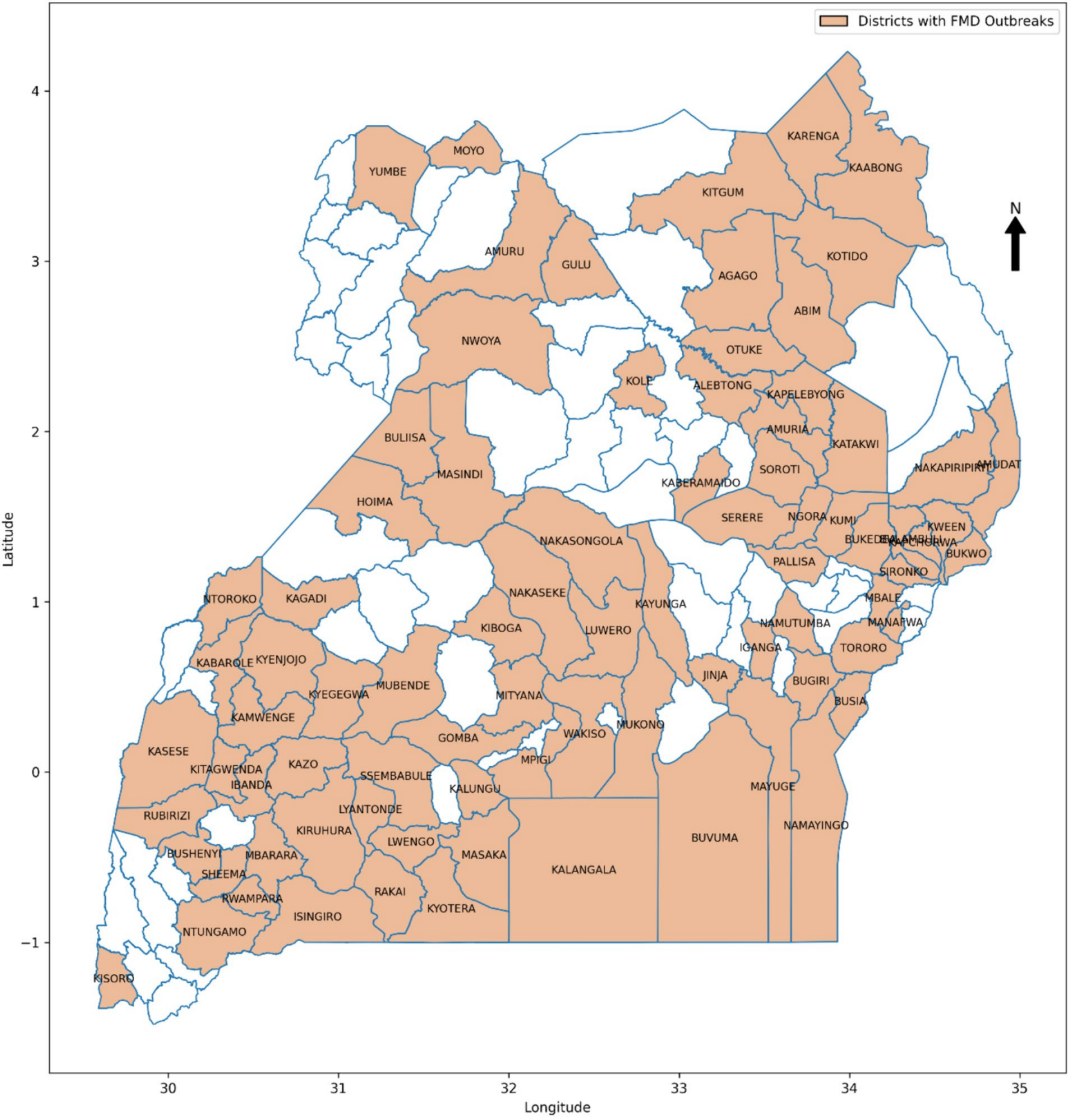
Prevalence of FMD outbreaks in Uganda between 2011 and 2022.

In the *endemic setting of Uganda*, FMD has persisted as a significant challenge for over 60 years (Munsey et al., 2019), leading to a 23% decline in income for livestock stakeholders at the processing level, along with reductions in market values of bulls and cows by 83 and 88%, respectively (Baluka, 2016). Despite implementing traditional intervention methods such as vaccination campaigns, quarantine measures, and movement restrictions, the country continues to face significant challenges in effectively mitigating the impact of FMD (Kerfua et al., 2018; Mwiine et al., 2019; Velazquez-Salinas et al., 2020). Figure 1 shows FMD prevalence between 2011 and 2022 across the districts of Uganda. The insufficient preparedness, partly due to lack of timely and accurate information on potential outbreaks, hinders the country’s response efforts (Munsey et al., 2019; Mwiine et al., 2019). The absence of such information undermines continuous monitoring of FMD for early detection and efficient distribution of resources, thereby greatly affecting the overall effectiveness of FMD control efforts (Munsey et al., 2019). This obstacle obstructs the country’s progress within the global Progressive Control Pathway for Foot and Mouth Disease (PCP-FMD) framework, aimed at assisting endemic countries reduce the impact of FMD by progressively increasing the level of control through development of risk-based control strategies (Sumption et al., 2012). The country remains at stage 2 of the 5 PCP-FMD framework (FAO, 2018), where early-warning systems are recommended for enhancing preparedness through continuous surveillance, enabling early detection of FMD and optimal resource allocation (Munsey et al., 2019).

Enhancing Foot and Mouth Disease (FMD) preparedness is essential to mitigate the impact of outbreaks (Yadav et al., 2020). Machine learning (ML)-based predictive modeling has shown promise in enabling early detection and optimal resource allocation for outbreak prevention and control (Punyapornwithaya et al., 2022). However, these models have been typically trained and tested in stationary environments where training and test data distributions are similar (Punyapornwithaya et al., 2022; Sueabua and Seresangtakul, 2023), neglecting the effects of varying distributions on predictive performance. The lack of empirical evidence on how ML models for FMD outbreaks perform under varying conditions presents a significant research gap. This gap is critical for policy makers in dynamic settings of Uganda, where key risk factors including rainfall and temperature (Munsey et al., 2019), influenced by climate change (Nsubuga and Rautenbach, 2018), exhibit distribution variability. Additionally, FMD outbreak data and influencing factors are dispersed across multiple sources (Kerfua et al., 2018; Obubu et al., 2021), complicating the creation of comprehensive and high-performing predictive models. This study aims to fill these gaps by (1) creating a unified and curated FMD dataset for Uganda, and (2) assessing the predictive performance degradation rates of ML models under varying distributions. The study makes several significant contributions:

- Provides a valuable unified dataset for future research.
- Offers insights into the impact of varying distribution on ML model performance, underscoring the need for adaptive approaches in changing environmental conditions.

The rest of the paper is structured as follows: Section 2 details comprehensive literature; Section 3 focuses on the methodology; Section 4 presents the study results; Section 5 discusses the findings; and Section 6 provides conclusions for the study.

## 2 Literature review

In this section, the study reviews related literature on the key factors influencing FMD in Uganda and across the African continent, identifies data sources, and examines prior research on the application of ML algorithms in predicting FMD for improved preparedness.

### 2.1 Risk factors influencing FMD outbreaks

The disease transmission occurs through contact with infected animals, secretions, or contaminated environments, as well as through aerosols, facilitating long-distance spread (Paton et al., 2018; Poonsuk et al., 2018; Brown et al., 2022). Contact with wildlife is another risk factor for FMD occurrence (Munsey et al., 2019). While the African buffalo, *Syncerus caffer*, is the only confirmed wildlife reservoir (Dubie and Negash, 2021), transmission occurs when livestock share grazing land or water points with wildlife, especially during the dry season when pastures and water become scarce (Miguel et al., 2017). Similarly, several studies, including Hamoonga et al. (2014), Hasahya et al. (2023), Jemberu et al. (2016), Munsey et al. (2019), and Sinkala et al. (2014), have stressed the significance of animal movements in disease spread. Additionally, research by Chimera et al. (2022), Dubie and Negash (2021), Fasina et al. (2013), Hamoonga et al. (2014), Jemberu et al. (2016), Jenbere et al. (2011), and Munsey et al. (2019) has highlighted the impact of animal density and demographics on transmission dynamics. Furthermore, environmental conditions, including temperature, and rainfall play a crucial role in FMD outbreaks, as shown by studies conducted by Ayebazibwe et al. (2010), Baluka et al. (2013), Molla et al. (2013), Hamoonga et al. (2014), Wungak et al. (2016), Abdela (2017), Munsey et al. (2019), Udahemuka et al. (2020), Kerfua et al. (2021), and Chimera et al. (2022). FMD impacts approximately 77% of the global livestock population (Bachanek-Bankowska et al., 2018; Zewdie et al., 2023), with seven known serotypes of the FMD virus: A, O, C, Asia 1, SAT 1, SAT 2, and SAT 3, causing varying distributions across regions (Jamal and Belsham, 2018; Paton et al., 2021; Salem et al., 2021). The low-income and middle-income countries bear 75% of the costs associated with preventing and controlling FMD, with Africa and Eurasia accounting for 50 and 33% of the total expenses, respectively (World Organization for Animal Health, 2024).

### 2.2 Absence of a unified and curated FMD dataset for Uganda

Historical FMD data for Uganda, collected over the past 60 years, is stored at the National Animal Disease Diagnostic and Epidemiology Centre (NADDEC) and the World organization for Animal Health (WOAH). This data includes key features such as the time and location of outbreaks, confirmed cases, animals at risk, and total animal density. Data on risk factors reported to influence FMD occurrences, such as rainfall and temperature, are maintained by the Uganda National Meteorological Authority (UNMA). Additional factors, including proximity to protected areas and international borders, can be accessed from various sources including the Pennsylvania State University. Despite prior literature identifying these critical factors (Ayebazibwe et al., 2010; Baluka et al., 2013; Abdela, 2017; Munsey et al., 2019; Kerfua et al., 2021), there remains a lack of a comprehensive, integrated, and curated dataset for predicting potential FMD outbreaks in Uganda. The existing data is fragmented across multiple organizations, hindering the development of effective predictive models. Therefore, this study aims to access data on historical FMD outbreaks and relevant risk factors, preprocess and integrate them into a unified and curated dataset. This dataset will be used for training, testing, and validating ML-based models to predict FMD outbreaks in Uganda. By creating a comprehensive dataset, the study seeks to enhance the performance and reliability of predictive modeling, ultimately improving FMD preparedness and response strategies in the country.

### 2.3 Machine learning-based prediction of diseases under stationary environment

In disease prediction, machine learning approaches are increasingly utilized across diverse fields. Uddin et al. (2019) conducted a comprehensive literature review encompassing various studies that examined supervised learning methods including Logistic Regression (LR), Decision Trees (DT), Random Forest, Support Vector Machines (SVM), Naïve Bayes, K-nearest neighbors (kNN), and Artificial Neural Networks (ANN) for predicting diseases including heart disease, diabetes, Parkinson’s disease, and breast cancer. Their focus centered on studies employing multiple supervised machine learning algorithms within the same research context for disease prediction. Their findings highlighted the frequent application of the Support Vector Machine algorithm in 29 studies and the Naïve Bayes algorithm in 23 studies. However, despite this prevalence, the Random Forest algorithm demonstrated notably higher performance. Among the 17 studies employing Random Forest, it exhibited the highest accuracy in 53% of cases, surpassing SVM, which achieved the highest accuracy in 41% of the studies it was involved in.

In another study, Carslake et al. (2020) leveraged machine learning and wearable sensor technology to monitor multiple behaviors in pre-weaned dairy calves. Through an AdaBoost ensemble learning algorithm, the research achieved high performance in identifying behaviors including locomotor play, self-grooming, feeding, and lying activity. Additionally, the study introduced an adjusted count quantification method specifically tailored to estimate the prevalence of locomotor play behavior. While showcasing substantial accuracy in behavior identification up to (99.73%), the quantification estimates revealed a notable correlation with the true prevalence of behaviors, albeit with a slight overestimation around (18.97%). This novel approach utilizing machine learning for behavior identification and quantification in calves using wearable sensors offers significant potential to assess calf health and welfare.

**Figure 2:**
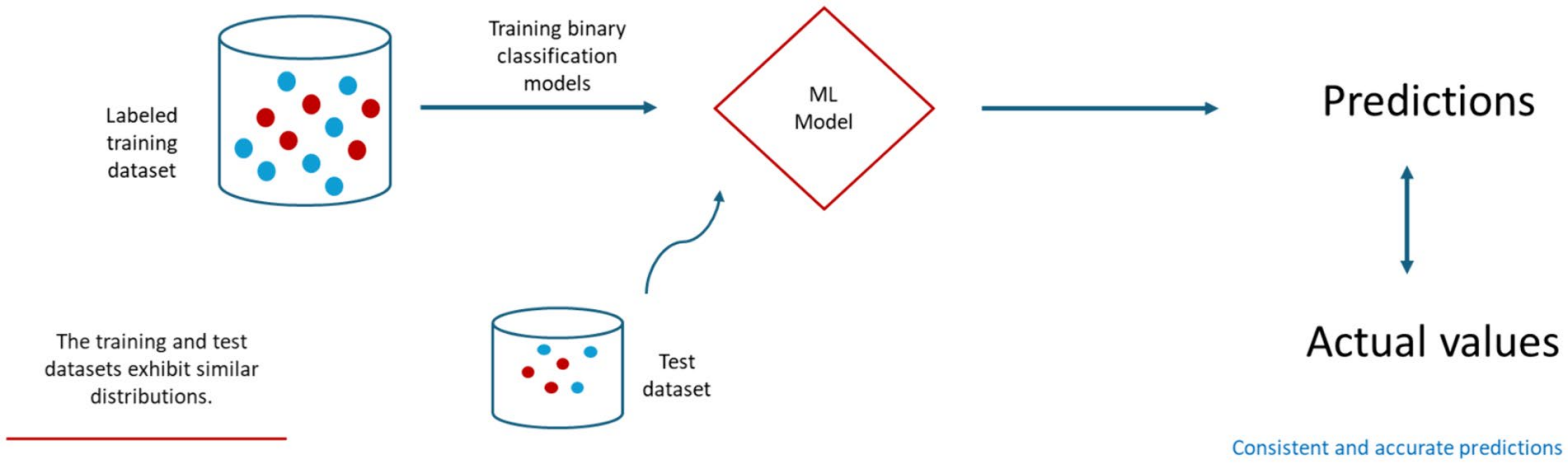
A general framework for training and testing ML-based prediction models for FMD under stationary environment.

In the prediction of FMD outbreaks for enhanced preparedness, Punyapornwithaya et al. (2022) explored ML algorithms to identify FMD outbreaks in the endemic setting of Thailand. In their study, algorithms, including classification trees, random forests, and Chi-squared automatic interaction detection (CHAID), were equipped with external risk factors as input variables. The results of the study were highly promising under stationary environment. Notably, the random forest model stood out, showcasing a remarkable predictive capability with an accuracy rate of 74%. Furthermore, it achieved the highest area under the operating characteristic curve (AUC) at 0.83 as shown in Table 1. Similarly, another study conducted by Sueabua and Seresangtakul (2023) also utilized machine learning techniques, such as support vector machines, decision trees, and artificial neural networks, to predict FMD outbreaks in the Nakhon Ratchasima province of Thailand. This research employed risk factors like rainfall, temperature, animal purchases in an animal market, sick animals in the month, and the percentage of vaccinated animals as input variables in the model development process. To address imbalanced datasets, the researchers applied the synthetic minority oversampling technique (SMOTE) to oversample the minority class, a common approach to mitigate class imbalance. The experimental results were quite promising, with the decision tree model adjusted for the imbalanced data, outperforming other models with an impressive accuracy rate of 98.86% as indicate in Table 1. However, like the previous study, the evaluation was based on a test dataset with a distribution similar to the training dataset as illustrated in Figure 2.

**Table 1:** Previous studies on machine learning-based prediction of FMD.

		Machine learning models			Prediction		Environment		
(Authors)	Region	Algorithms	Best model	Accuracy	Outbreaks	Severity	Stationary	Dynamic condition	Risk factors
Punyapornwithaya et al. (2022)	Chiang Mai Province, Thailand	Classification Tree, Random Forest, and Chi-Squared Automatic Interaction Detection	Random Forest	74%	✔	x	✔	x	Purchasing, grazing, roughage source, farm visited by artificial insemination technicians, neighbors of an FMD outbreak farm, manure trading, farm experienced FMD, slaughterhouses, close to the main road, farm located near live cattle market, visiting live cattle market, distance to the closest cattle farm, vaccinations, utilization of water from natural sources.
Sueabua and Seresangtakul (2023)	Nakhon Ratchasima Province, Thailand	Support Vector Machine, Decision Trees, and Artificial Neural Networks (ANN)	Decision Tree	98.86%	✔	x	✔	x	Rainfall, temperature, animal purchases in an animal market, sick animal in the month, percent of vaccinated animals

The findings emphasize machine learning models’ vital role in predicting outbreaks for better disease management. However, their reliance on the independently identically distributed data assumption creates vulnerability to distribution shifts, limiting their use in new environments and affecting global disease mitigation efforts. Despite previous studies exploring machine learning for FMD prediction, they often overlooked varying distributions, a known concern in the field. The potential occurrence of these distribution variability over time may affect model performance, making them unreliable for FMD outbreak prediction. Given the disease’s rapid spread and impact on the livestock industry, timely intervention is crucial. Therefore, investigating distribution shifts in FMD datasets and their impact on machine learning methods is critical in developing adaptive models capable of accurate predictions, enhancing preparedness against FMD’s rapid spread.

## 3 Materials and methods

### 3.1 Utilizing the experimental design to conduct the study

**Figure 3:**
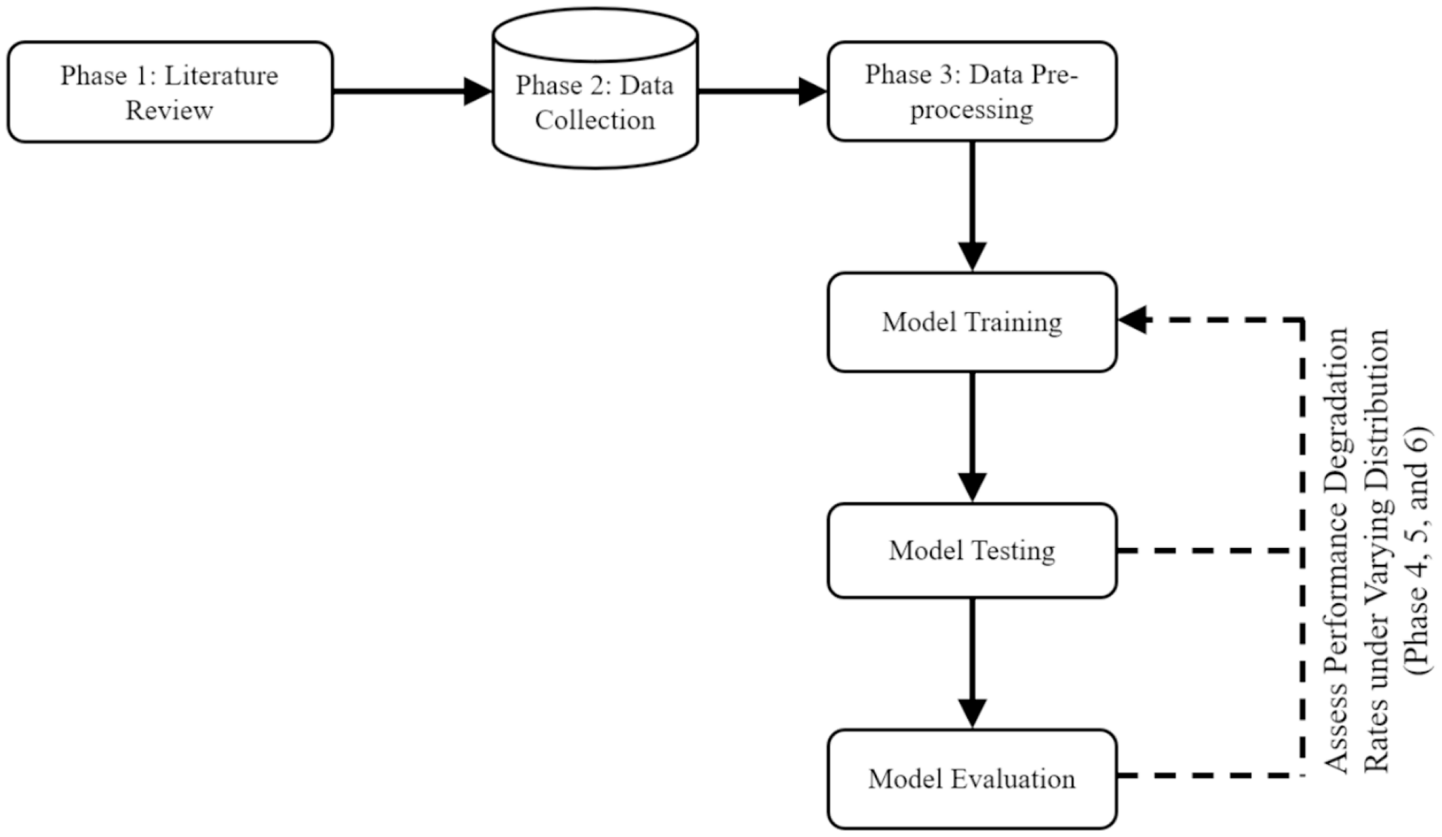
An experimental methodology to guide the study.

To achieve the research objectives of developing a unified and curated FMD dataset and assess predictive performance degradation rates under varying distributions in Uganda, the study adopted an experimental research design. Experimental research design in ML entails a systematic methodology for planning, executing, and analyzing experiments to assess the performance of ML models while minimizing biases, noise, and distribution mismatch (Kamiri and Mariga, 2021). By adhering to a well-defined experimental design, the study can make informed decisions regarding the ML models, leading to improved performance and a deeper comprehension of the underlying mechanisms. Figure 3 outlines the experimental methodological approach with various phases for conducting experiments to meet the specified objectives in this study. The phases include Literature review, Data collection, Data pre-processing, Model training, Model testing, and Model evaluation.

In this study, six phases depicted in Figure 3 were carried out across six key activities. Phase 1 (section 3.1.1) encompasses the activities of identifying a research problem and data sources. Phase 2 (section 3.1.2) focuses on data acquisition and compilation. Phase 3 (section 3.1.3) involves data cleaning and integrating disparate datasets into a unified and curated dataset. Phase 4 (section 3.1.4) involves training seven ML-based models. Phase 5 (section 3.1.5) entails testing the predictive performances of the seven trained models. Phase 6 (section 3.1.6) focuses on evaluating the predictive performances of the trained seven models using validation set. Table 2 provides a summary of these phases, key activities, accomplished study objectives, methods, and descriptions illustrating how the methods were employed to achieve the objectives. In the following sections, the study discusses in detail how the various phases are executed to achieve the study objectives.

**Table 2:** A summary of the research phases, objectives, and methods for achieving the research objectives.

Phase No.	Activity	Study objective	Research methods	Description
1	Identification of the problem and data sources	–	Literature review	The research problem was conceptualized through a traditional review of literature on existing methods known to address DS in the ML domain. Their failure when DS is significant and inconsistency in performance across various datasets were evident. Furthermore, through a literature review guided by PRISM, the study identified key risk factors that influence FMD outbreaks, which were used to identify the data sources for the collection of datasets. They also acted as predictors in developing the ML-based model for predicting FMD outbreaks in Uganda.
2	Data collection	–	Retrospective	A retrospective approach was adopted to guide the collection of historical data on FMD outbreaks and the identified risk factors including rainfall, temperature, proximity to international borders, adjacency to national parks, and cattle density.
3	Harmonizing disparate datasets for a unified and curated dataset	Objective 1	Experiments (pd.merge, imputation, drop_duplicates, and visualization)	To harmonize disparate datasets, the study performed data pre-processing by handling missing datapoints, duplicate records, outliers and integration of datasets into a unified and curated dataset
4, 5 and 6	Training seven ML models, Testing and Assessment of the predictive performance degradation rates in ML-based prediction of FMD outbreaks under varying distribution	Objective 2	Experiments (Model Training, testing, and evaluation of Performance)	Seven ML models were trained, tested, and subjected to the target dataset with varying distribution for evaluation. The performance degradation rates were computed to show the reduction in performance under distribution shifts using classification performance metrics including accuracy, AUC, recall, precision, and F1-score.

#### 3.1.1 Phase 1: literature review

In Phase 1, the study conducted a traditional literature review to identify the research problem and a systematic literature review, guided by the Preferred Reporting Items for Systematic Reviews and Meta-Analyses (PRISMA) framework, to identify the risk factors influencing FMD outbreaks, as discussed in the following sections.

##### 3.1.1.1 Identification of research problem and risk factors

**Figure 4:**
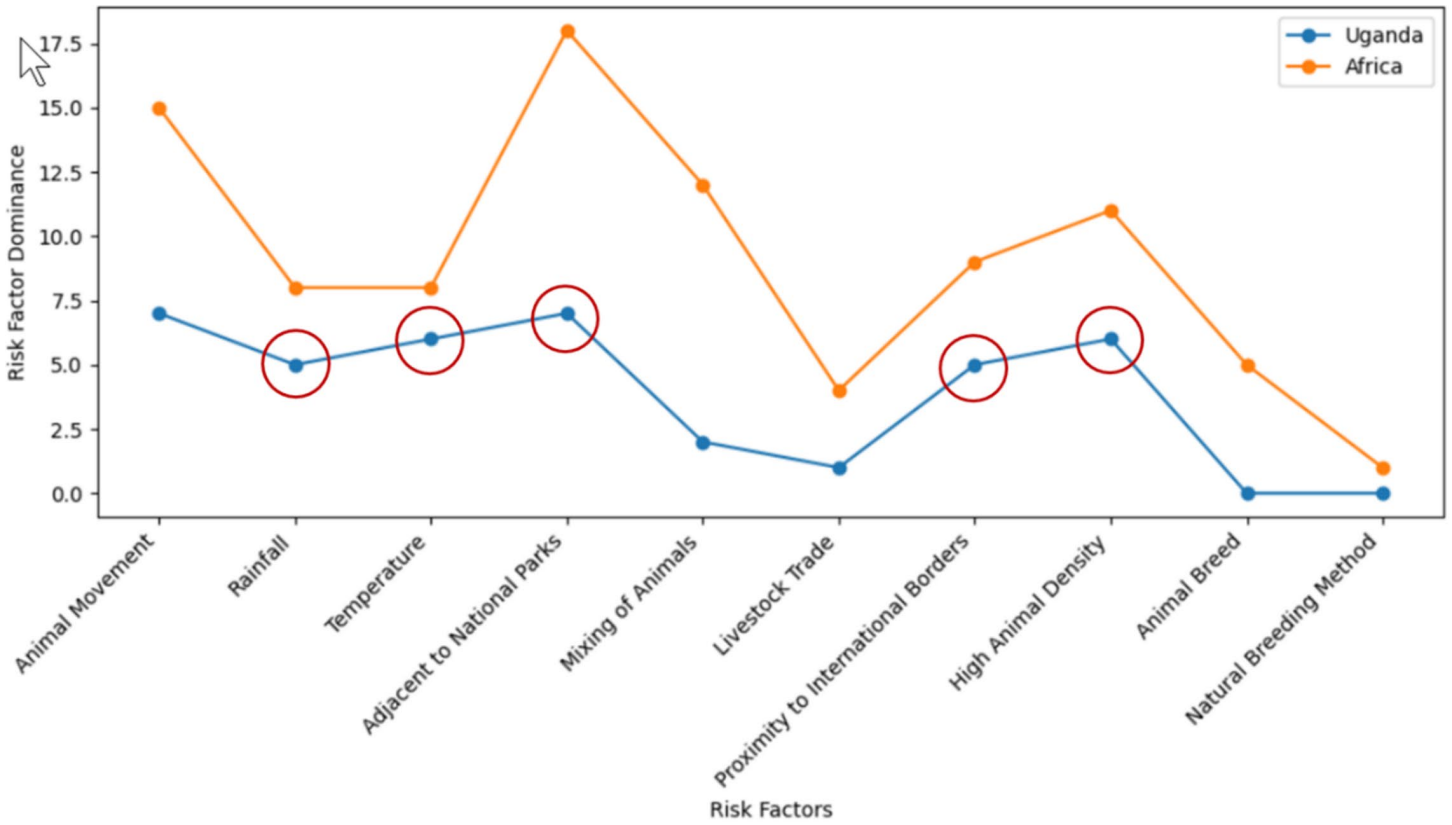
Visualization of FMD risk factors across Africa and the specific setting of Uganda.

This phase largely involved conducting a traditional and systematic literature review, as reported in section 2 Literature Review. The study highlighted the inadequacy in FMD preparedness and the potential of ML to generate predictive information for continuous surveillance, enabling early detection and optimal allocation of resources. Furthermore, the study identified absence of a unified and curated FMD dataset as an obstacle in developing ML-based predictive models. Additionally, the study identified the uncertainty aspect on the extent of performance degradation that can be caused by varying distribution in key risk factors including rainfall and temperature. While previous research has made significant advancements in ML-based prediction of FMD outbreaks (Punyapornwithaya et al., 2022; Sueabua and Seresangtakul, 2023), the focus on stationary environments renders such predictions vulnerable to unexpected distribution shifts. Therefore, this study investigated the extent to which varying distribution can have on the ML-based predictive performance for FMD outbreaks in Uganda. Based on this research gap identified through comprehensive literature review, research objectives were formulated. In addition, this phase identified crucial risk factors influencing FMD outbreaks, such as rainfall, temperature, proximity to protected areas, proximity to international borders, and cattle density (Figure 4). The circled factors (Figure 4) were utilized to identify data sources (Table 3) and acted as predictors in constructing the predictive model. Furthermore, the study utilized a descriptive statistical approach, leveraging Python 3.11.4 with Jupyter Notebook 7.0.0. Python libraries, particularly Pandas and Matplotlib, were instrumental in data handling, analysis, and visualization.

**Table 3:** FMD risk factors and data sources.

Content	Source	Timeframe
Historical data on monthly FMD outbreaks [*location, time, vaccination coverage, serotype, confirmed cases, animal at risk*]	NADDEC, WOAH	2011–2022
Climate data [*rainfall, temperature*]	UNMA, https://iridl.ldeo.columbia.edu	2011–2022
Animal density	NLC2008, MAAIF & UBOS	
Adjacent to protected areas	Pennsylvania State University Department of Geography psugeo.org/Africa/Africa_files/	
Adjacent to international border	Pennsylvania State University Department of Geography psugeo.org/Africa/Africa_files/	

#### 3.1.2 Phase 2: data collection

Uganda, situated in East Africa, is a landlocked country bordered by Kenya to the east, Tanzania to the south, Rwanda to the southwest, South Sudan to the north, and the Democratic Republic of Congo to the west. Positioned near the equator, Uganda spans diverse landscapes, encompassing expansive savannahs, dense forests, and the towering Rwenzori Mountains. With a latitude range of approximately 1°N to 4°N and a longitude between 29°E and 35°E, Uganda experiences a tropical climate, fostering a wide array of flora and fauna. The country’s diverse topography and climates contribute to varied ecological conditions, potentially affecting disease transmission dynamics (Munsey et al., 2019). From the lush vegetation of the southern regions to the arid landscapes in the north, these geographic and climatic diversities can significantly influence the occurrence and spread of FMD outbreaks, underscoring the importance of a comprehensive approach to disease prediction and control strategies within the country.

The study utilized a retrospective approach to guide the data collection process. The choice is justified by the approach’s ability to access historical information spanning a significant period, providing a rich dataset crucial for training and validating the ML-based predictive model for FMD. The study gathered an extensive dataset, spanning the period from 2011 to 2022 and encompassing various critical sources of information (Table 3). From 2011 to 2022, FMD outbreaks were confirmed in 86 districts across the country, as shown in Figure 1, with their prevalence detailed in Supplementary Figure 1. The historical FMD outbreak data were obtained from reputable sources, including the NADDEC and WOAH. The dataset contained essential details such as outbreak locations, timing of occurrence, and confirmed cases. Additionally, the study incorporated climatic factors by including rainfall and temperature data from the Uganda National Meteorological Authority (UNMA). Furthermore, to account for livestock-related factors, the utilized data from the National Livestock Census 2008 (NLC2008), jointly conducted by the Ministry of Agriculture, Animal Industry, and Fisheries (MAAIF) and the Uganda Bureau of Statistics (UBOS). The data provided valuable insights into livestock population densities across different regions. Moreover, the study collected geographical information concerning areas adjacent to protected wildlife zones and international borders from the Pennsylvania State University Department of Geography psugeo.org/Africa/Africa_files/, as these geographical features significantly influence FMD transmission dynamics. The FMD risk factors and their corresponding data sources are shown in Table 3.

##### 3.1.2.1 Data sampling

**Figure 5:**
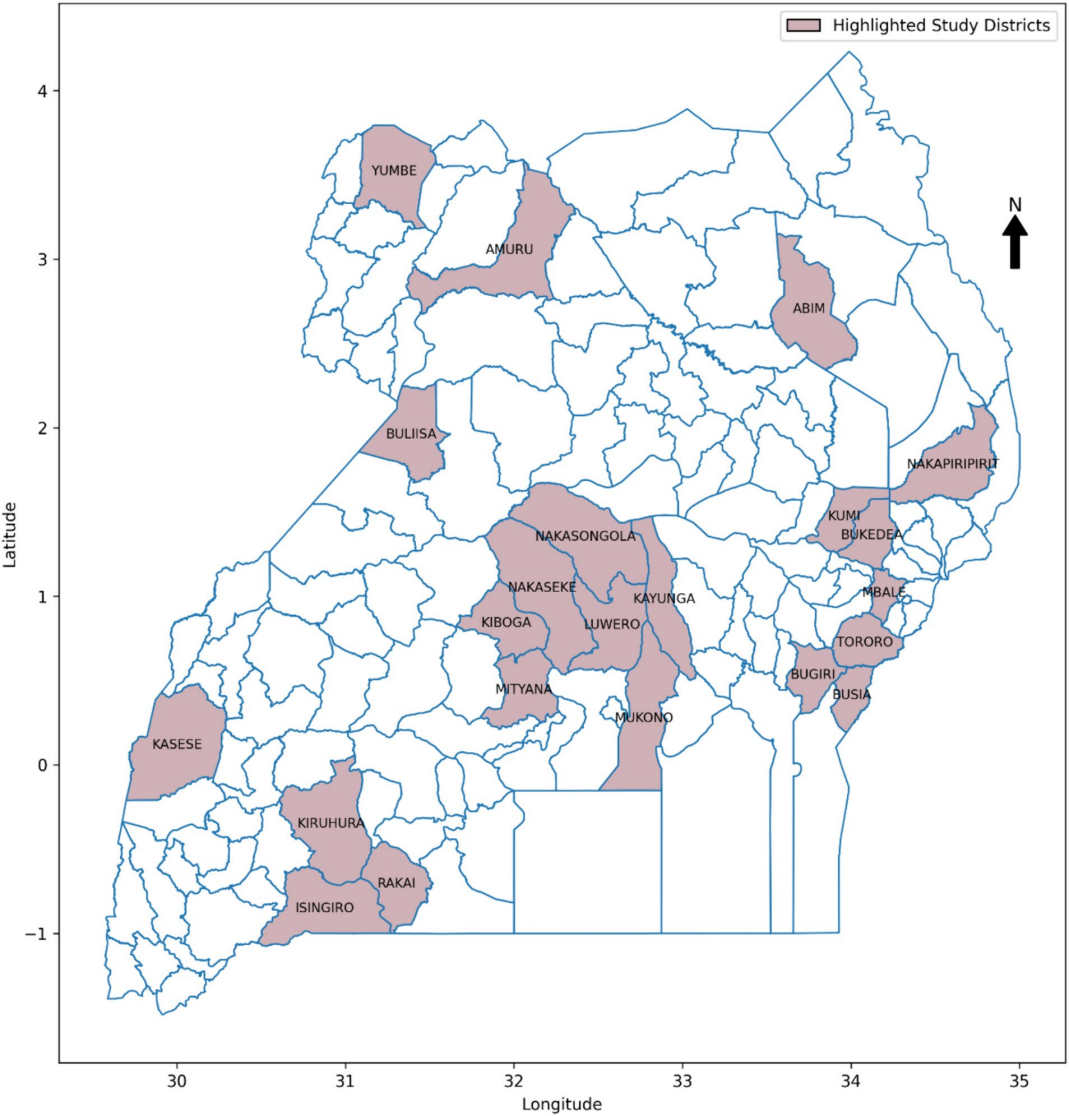
Map of Uganda with highlighted study districts.

Various data sampling techniques exist in data science, each suitable for different research needs (Bhardwaj, 2019; Sarker and Al-Muaalemi, 2022). This study leveraged the insights gained from the dominance of FMD outbreaks across the districts of Uganda, as illustrated in Supplementary Figure 1, 22 districts were purposively selected for inclusion (Figure 5). Specifically, the circled districts with the highest frequency of outbreaks during the study period of 2011–2022 were prioritized (Supplementary Figure 1). This approach ensured that the ML-based models had access to a substantial amount of data, which is crucial for their performance. Additionally, it helped avoid high imbalanced datasets that could negatively impact ML performance, especially in districts with fewer outbreaks. Moreover, research indicates that dominant districts, often referred to as hotspots, serve as sources of outbreaks that spread to other districts. Therefore, by focusing on these dominant districts, the study aimed to facilitate generalization to other districts and enhance the predictive model’s applicability.

#### 3.1.3 Phase 3: data pre-processing

**Figure 6:**
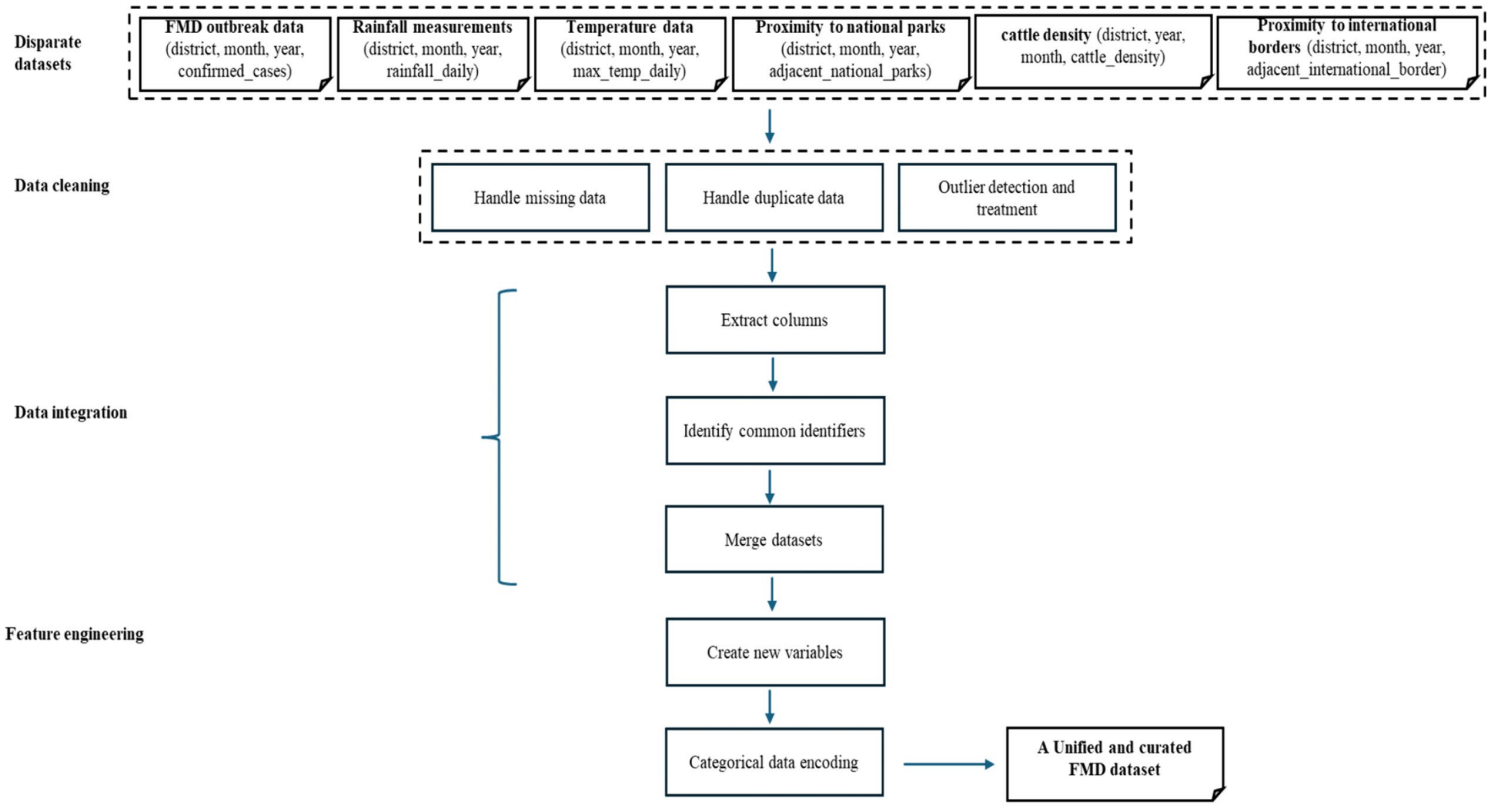
Visualization of the data pre-processing workflow.

In Phase 3, the study aimed to achieve a unified and curated dataset for training, testing, and evaluating ML-based predictive models for FMD outbreaks in Uganda. Data pre-processing is a crucial step in ML-based research, focused on refining and harmonizing datasets from various sources (Grafberger et al., 2021; Laila et al., 2022). This preparatory phase ensures data accuracy and reliability by rectifying inconsistencies, eliminating redundant information, and addressing missing or erroneous data entries. Additionally, it establishes uniformity across disparate datasets, facilitating seamless integration and analysis. The pre-processing workflow is illustrated in Figure 6.

##### 3.1.3.1 Handling missing values

During data preprocessing, addressing missing values from various sources, including historical FMD outbreak datasets and environmental data, was crucial. Imputation techniques were employed to handle these gaps, with mean imputation being the chosen strategy (Ahn et al., 2022). Python, with libraries like Pandas, offered effective tools for identifying missing values. Functions including isnull or isna along with methods like sum facilitated the assessment of missing data prevalence per feature in datasets. For instance, using df.isnull().sum with a Pandas DataFrame ‘df’ efficiently detected missing values across columns. Mean imputation involved substituting missing values with the mean of their respective features. This approach aimed to maintain dataset completeness and preserve critical variables necessary for subsequent analyses and model development. The datasets retained essential information by employing mean imputation, ensuring integrity for analyzing risk factors associated with FMD outbreaks in Uganda. This strategy prevented the loss of valuable data points, enabling comprehensive analyses and robust model development with a more complete dataset.

##### 3.1.3.2 Handling duplicate records

Addressing duplicate records from various datasets, including FMD outbreak historical records and environmental data, was crucial during preprocessing. Removing duplicates aimed to eliminate redundancy and ensure data accuracy (Tae et al., 2019; Mishra et al., 2020). Python, with libraries like Pandas, facilitated efficient detection and elimination of duplicate records. The drop_duplicates() function in Pandas allowed for the identification and removal of duplicate entries from a DataFrame. For instance, using df.drop_duplicates(subset = [‘column1’, ‘column2’], keep = ‘first’, inplace = True) with a Pandas DataFrame ‘df’ enabled the detection and deletion of duplicate entries based on specified columns.

Eliminating duplicates was vital for dataset accuracy and integrity. Duplicate entries could introduce biases, skewing analytical outcomes and affecting modeling reliability. Removing duplications preserved dataset integrity, ensuring each entry was unique and meaningful to analysis. This process enhanced dataset quality by ensuring each record was distinct and accurate, minimizing the risk of inflated statistics or biased outcomes. Removing duplicate records refined the dataset, laying the groundwork for more accurate analyses and predictive performances for FMD outbreaks in Uganda.

##### 3.1.3.3 Outlier detection and treatment

Outlier detection and treatment were essential during preprocessing to ensure data consistency and accuracy. Python, with libraries like Pandas, Matplotlib, and NumPy, provided robust techniques for this task. The Z-score method was effective for identifying outliers, calculating the deviation of a data point from the mean in terms of standard deviations. Points with Z-scores beyond a threshold were considered outliers (Chikodili et al., 2021). Scatter plots visually confirmed identified outliers, aiding in recognizing data points significantly deviating from the general pattern.

Once outliers were confirmed, mean imputation treated them by replacing outlier values with the mean of the respective feature. Despite more sophisticated methods available, mean imputation was chosen for its simplicity and effectiveness in maintaining data consistency and integrity (Rubin, 2018; Jadhav et al., 2019). This meticulous outlier detection and treatment resulted in a refined dataset, devoid of extreme values that could skew analytical processes. By ensuring data integrity, we enhanced the accuracy and reliability of subsequent analyses and the FMD outbreak prediction model. This process was critical in training the model on accurate data, leading to more precise and dependable predictive modeling of FMD outbreaks.

##### 3.1.3.4 Data integration

**Figure 7:**
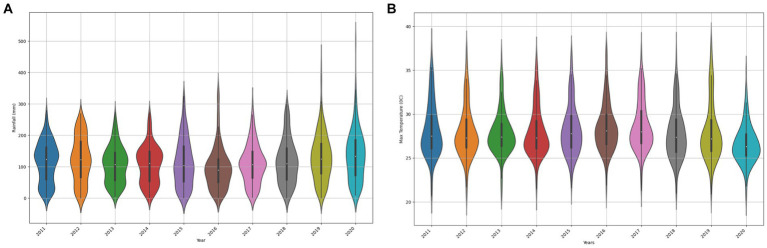
Visualization illustrating the variability in rainfall **(A)** and max temperature **(B)** features, highlighting distribution shifts.

Data integration involves merging multiple datasets from various sources into a unified and coherent dataset (Isaac et al., 2020). Creating a comprehensive dataset that consolidates information from different sources is crucial for enabling more effective and thorough analysis. By utilizing the set function in Python, the study extracted columns from disparate datasets loaded in DataFrames, including historical FMD outbreak records (district, month, year, confirmed_cases), rainfall measurements (district, month, year, rainfall_daily), temperature data (district, month, year, max_temp_daily), proximity to national parks (district, month, year, adjacent_national_parks), cattle density (district, year, month, cattle_density), and proximity to international borders (district, month, year, adjacent_international_border) into sets. Using the intersection function on these sets, common columns were identified. Finally, the Pandas pd.merge function was utilized to combine the datasets based on the identified common identifiers. The choice of using the pd.merge function in Python is justified by its flexibility in merging datasets based on common features. It allows for a seamless integration process, ensuring that relevant information from different datasets is appropriately combined. The flowchart (Figure 6) illustrates this integration process, guiding the sequential steps from extracting columns into sets, identifying common identifiers, to joining datasets, ensuring consistency and reliability in preparing the unified dataset. The data distribution of the unified and curated dataset in shown in Figure 7.

##### 3.1.3.5 Feature engineering

Feature engineering, a vital process in data pre-processing, involves creating new variables or modifying existing ones to enhance the performance of machine learning models (Kang and Tian, 2018; Maharana et al., 2022). It transforms raw data into meaningful features that better represent the underlying problem. In this study, feature engineering was utilized to create two key features: monthly rainfall (rainfall) and monthly maximum temperature (max_temp). Daily rainfall measurements were averaged, and daily maximum temperature values were averaged to align with the monthly FMD outbreak data. These engineered features were crucial for improving the predictive accuracy of the models, allowing for a more relevant and effective analysis of FMD outbreaks.

##### 3.1.3.6 Categorical data encoding

During data preparation, categorical data encoding was crucial for converting qualitative variables into numerical formats, essential for machine learning algorithms (Jo, 2021). Using Pandas in Python, a ‘target’ class was created to represent outbreak (1) and non-outbreak (0) instances. This encoding was achieved by mapping ‘outbreak’ to 1 and ‘no-outbreak’ to 0 in the ‘target’ column using Pandas’ map() function. Converting categorical variables into numerical representations facilitated machine learning models’ interpretation of outbreak occurrences, aiding in predictive modeling (Hancock and Khoshgoftaar, 2020).

#### 3.1.4 Phase 4: model training, testing and evaluation

In Phase 4, the study, aimed to investigate the performance degradation rates of ML-based models in predicting FMD outbreaks in the dynamic setting of Uganda. To fulfill this objective, the study conducted experiments on selected ML algorithms known to exhibit better predictive power using supervised learning techniques. In the following sections, study provides a detailed discussion of the methods employed to achieve this objective.

##### 3.1.4.1 Splitting the dataset into reference (training) and current (target) sets

While investigating the degradation in performance exhibited by ML-based algorithms in a non-stationary environment when predicting FMD outbreaks using a curated dataset, a pivotal step entailed dividing the dataset into two subsets: the reference and target datasets. This study employed the sequential sampling technique which is suitable for splitting timeseries data. The reference (training) dataset encapsulated records from 2011 to 2020, while the current (target) dataset encompassed records from 2021 to 2022. This division allowed for distinct periods for training and validation purposes, ensuring that the models developed were based on historical data reference and then validated against more recent target information.

##### 3.1.4.2 Visualizing varying distributions for rainfall and max temperature

To confirm data variability in rainfall and Max Temperature features, the study utilized violin plots to depict the changes in rainfall and max temperature distributions across different years in Uganda. Variations in the shape, median position, and quartiles within each violin plot highlight the dissimilarities in rainfall distribution patterns over time. The choice to use rainfall and temperature as metrics for demonstrating potential distribution shifts is grounded in the literature indicating their significance as contributing factors to FMD outbreaks in Uganda (Munsey et al., 2019).

##### 3.1.4.3 Handling class imbalance

**Figure 8:**
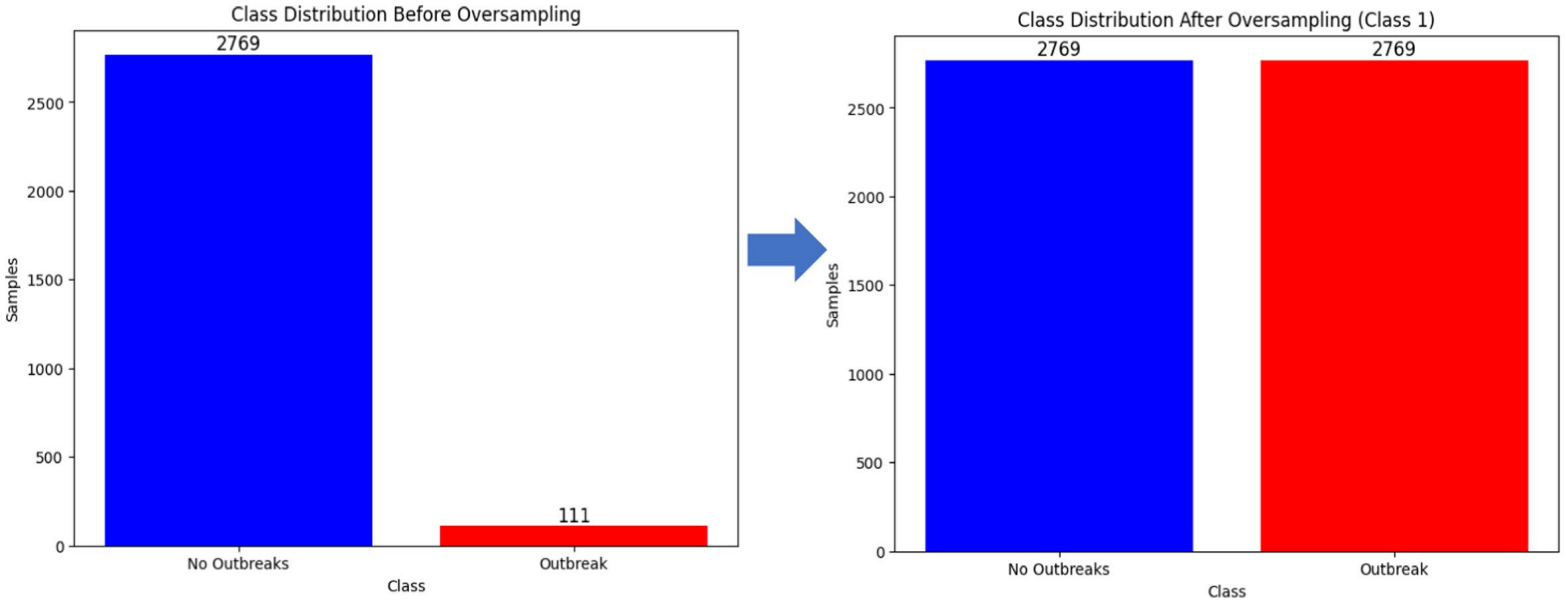
Utilizing SMOTE (original), SMOTE-SVM, borderline-SMOTE and ADASYN techniques for oversampling the minority class for a balanced dataset.

In ML domain, various researchers have reported on the significant impact of class imbalance on the predictive performance of ML-based algorithms (Tran et al., 2021). Class imbalance is a phenomenon where the instances of one class (majority) are significantly more than the samples in the other class (minority) (Buda et al., 2018). The study explored matplotlib to visualize the distribution of the dataset. Supplementary Figure 2 confirms the existence of existence of class imbalance where the non-outbreak samples (majority) are significantly more than the outbreak samples (minority). To a certain the impact of class imbalance, the study trained baseline models with the imbalanced instances. Additionally, the study conducted a random under-sampling technique to balance the classes (Supplementary Figure 3) and evaluated the performance. Similarly, the study explored SMOTE (original) and its variants including borderline-SMOTE, SMOTE-SVM, and ADASYN techniques to mitigate the impact of imbalanced classes (Supplementary Figure 4; Figure 8). The choice of SMOTE and its variants over other augmentation techniques is justified by its ability to intricately handle imbalanced datasets. Unlike random oversampling or under-sampling methods, SMOTE generates synthetic instances by considering the attributes of existing data points, thus producing more diverse and representative samples.

##### 3.1.4.4 Experiments to assess performance degradation rates under distribution shifts

**Figure 9:**
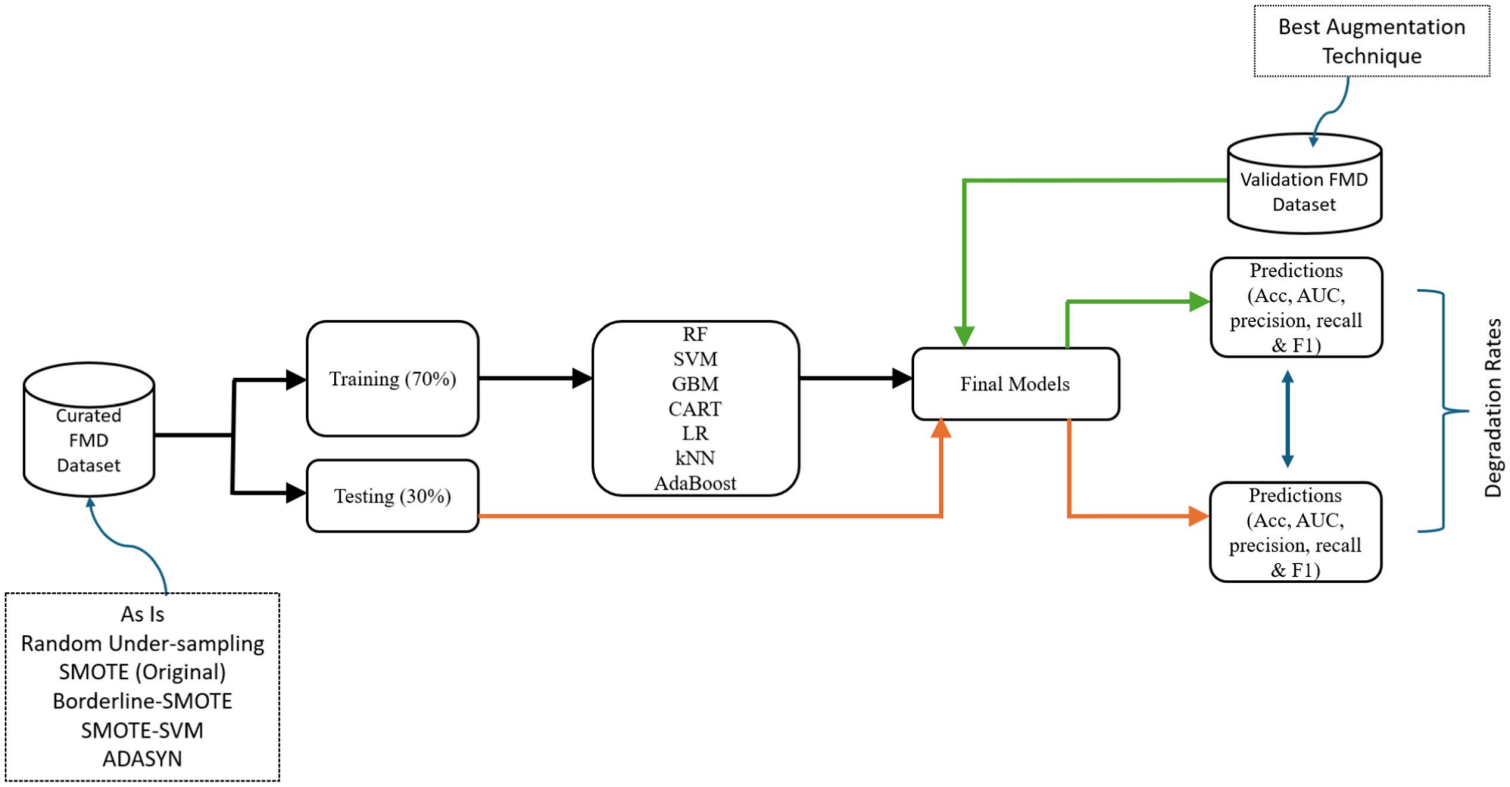
Experimental flowchart for the model development process.

To assess the performance of ML-based models in predicting FMD outbreaks under distribution shifts required selecting appropriate ML algorithms suitable for the FMD datasets. In the subsequent sections the study discusses the experimental setup where seven ML algorithms were chosen, trained, tested and validated their performances using target dataset (Figure 9).

The pipeline guides the development of Random Forest (RF), Support Vector Machine (SVM), Gradient Boosting Machine (GBM), Classification Regression Tree (CART), Logistic Regression (LR), k-Nearest Neighbors (kNN), and AdaptiveBoost (AdaBoost) models, testing, and validation.

###### 3.1.4.4.1 Experimental setup

The study designed experiments for developing and evaluating ML-based models for predicting FMD outbreaks under non-stationary environment. The study began by employing Python programming language 3.11.4, known for its rich collection of libraries and tools tailored explicitly for machine learning applications (Soklaski et al., 2022; Rajamani and Iyer, 2023). This choice facilitated the data analysis and development processes, allowing efficient data exploration and code development within the Jupyter Notebook integrated development environment (IDE) 7.0.0 (Brewer et al., 2022; Hewage and Meedeniya, 2022).

In optimizing the computational resources, the study relied on a local machine learning platform configured to synergistically utilize the Graphics Processing Unit (GPU) and Central Processing Unit (CPU). Leveraging this combined processing power significantly accelerated general-purpose machine learning tasks, expediting the research pace and productivity. The study employed Pandas library for effective data manipulation, which excels in handling diverse data formats and structures (Chang et al., 2022). Complementing this, the study utilized NumPy for numerical operations and array manipulation, acknowledging its fundamental role in data science and machine learning (Ziatdinov et al., 2022; Rajamani and Iyer, 2023).

The development and evaluation of the ML-based models were conducted using the Scikit-Learn library (Narayanan et al., 2022). This comprehensive library offered extensive machine learning algorithms and evaluation tools, streamlining the experimentation process. Additionally, the study employed Matplotlib and Seaborn for data visualization and result communication. These visualization libraries created insightful graphs and charts (Schessner et al., 2022; Weiss, 2022). This thoughtfully constructed environment and toolset played a pivotal role in establishing a robust foundation for model training and subsequent analyses. They ensured the reliability and validity of the research outcomes, providing a structured and efficient framework for experimentation and evaluation.

###### 3.1.4.4.2 Choosing machine learning algorithms

The selection of machine learning algorithms for predicting FMD outbreaks stemmed from the groundwork laid by Punyapornwithaya et al. (2022) and Sueabua and Seresangtakul (2023). Their research explored the efficacy of supervised learning methods in predicting FMD outbreaks within Thailand’s provinces. However, despite showcasing promising predictive capabilities, these prior studies overlooked the crucial aspect of assessing the models’ performance under distribution shifts, a significant limitation addressed in this research. By building upon this foundation, the researcher chose seven distinct machine learning algorithms for their proven strengths in predictive modeling (Karapapas and Goumopoulos, 2021; Dutta et al., 2022). The study chose Random Forest (RF), Support Vector Machine (SVM), k-Nearest Neighbors (kNN), Gradient Boosting Machine (GBM), AdaBoost, Logistic Regression (LR), and Classification and Regression Tree (CART) for predicting Foot and Mouth Disease outbreaks in the endemic settings of Uganda due to their diverse functionalities and strengths in handling various aspects of predictive modeling.

*Random Forest*: RF is a versatile ensemble learning method that excels in handling large datasets and complex interactions among variables (Choudhury et al., 2021). Its ability to aggregate the predictions of multiple decision trees reduces overfitting and enhances predictive performance.

*Support Vector Machine:* SVM is renowned for handling high-dimensional data and finding optimal hyperplanes for classification tasks (Cervantes et al., 2020). Its effectiveness in separating data points with a clear margin makes it suitable for binary classification problems like predicting FMD outbreaks.

*Classification and Regression Tree*: CART provides transparent decision-making processes through interpretable tree structures (Aghaei et al., 2021). Its simplicity and ease of interpretation make it a valuable tool for understanding the relationships between predictors and the target variable.

*Logistic Regression*: Logistic Regression, a classic method, remains robust and effective, especially in binary classification problems (Joshi and Dhakal, 2021). Its straightforward implementation and interpretability make it a staple in predictive modeling.

*Gradient Boosting Machine:* GBM is included due to its capability to effectively handle complex relationships in the data and its robustness against overfitting (Touzani et al., 2018). By building multiple weak learners sequentially, each learner focuses on the mistakes of its predecessors, leading to a strong overall model.

*k-Nearest Neighbors:* kNN is valuable in non-linear data scenarios by finding patterns based on neighboring data points (Bansal et al., 2022). Its simplicity and effectiveness in capturing local data patterns make it a useful addition.

*AdaBoost:* AdaBoost is a powerful ensemble learning technique that works by sequentially training a series of weak learners, such as decision trees, with each subsequent learner focusing on the examples that were misclassified by the previous ones (Mienye and Sun, 2022). Its robustness to overfitting and ability to generalize well to new data, along with its effectiveness in handling imbalanced datasets, make it a valuable tool for predicting FMD outbreaks.

The selection of these algorithms was grounded in their diverse functionalities, aimed at capturing various aspects of FMD outbreak prediction. Each algorithm brings unique capabilities, ensuring a comprehensive exploration of predictive modeling for FMD outbreaks, considering the details and complexities within the unified dataset. By focusing on these seven models, the study aimed to balance predictive power, interpretability, and computational feasibility in the context of predicting FMD outbreaks in Uganda under varying distribution.

####### 3.1.4.4.2.1 Selection criteria and hyperparameter tuning

The study employed RF, SVM, LR, GBM, AdaBoost, CART, and kNN for predicting FMD outbreaks in Uganda. The choice of RF, GBM, and AdaBoost was motivated by their strong ensemble predictive power (Sahin, 2020). SVM and LR were selected for their robustness in high-dimensional spaces (Pisner and Schnyer, 2020) and binary classification (Nusinovici et al., 2020), respectively, while kNN and CART were chosen for their simplicity and interpretability (Zafar and Khan, 2021). Moreover, these models exhibit computational efficiency in prediction (Reddy et al., 2020; Shobana and Umamaheswari, 2021; Singh et al., 2021; Sethuraman et al., 2023). The study used default hyperparameters across all models, justified by prior works (Punyapornwithaya et al., 2022; Sueabua and Seresangtakul, 2023) and to maintain consistency and simplicity in comparative analysis.

###### 3.1.4.4.3 Performance evaluation metrics

In this study, classification performance metrics were employed to assess the efficacy of the learning algorithms in predicting FMD outbreaks. From the literature, performance is evaluated using two data sets: the training and test or validation sets (Ferri et al., 2009; Jiao and Du, 2016; Tharwat, 2020). The robustness of the ML-based models utilized in the experiments was evaluated through various performance metrics that provide quantitative measures, including accuracy, F-score, recall, precision, and the Area Under the Curve (AUC) of the Receiver Operating Characteristic (ROC) curve. Below, the study elaborates on the formulas utilized to calculate these performance metrics.

*Accuracy (ACC):* Accuracy measures the overall correctness of predictions made by the model (El-Hasnony et al., 2022). It is calculated as the ratio of correctly predicted instances to the total instances.


$$ACC=\frac{TP+TN}{TP+TN+FP+FN}$$

TP (true positive) is the number of samples whose actual value is positive, and the model predicts them as positive. TN (true negative) is the number of samples whose actual value is negative, and the model predicts them as negative. FP (false positive) is the number of samples whose actual value is negative, and the model predicts them as positive. FN (false negative) is the number of samples whose actual value is positive, and the model predicts them as negative.

*Area Under the Curve (AUC) of the Receiver Operating Characteristic (ROC) Curve:* AUC measures the model’s ability to distinguish between positive and negative instances (Wu et al., 2020; Kaur et al., 2022). It quantifies the area under the ROC curve, where a higher AUC indicates better model performance.

Precision (PR): Precision assesses the proportion of true positive predictions among all positive predictions made by the model (Iwendi et al., 2020; Wang et al., 2020).


$$PR=\frac{TP}{TP+FP}$$

*Recall [Sensitivity (SE)] or True Positive Rate (TPR):* Recall measures the proportion of true positive predictions among all actual positive instances (Powers, 2020).


$$\mathrm{Recall}=\frac{TP}{TP+FN}$$

*F1-score:* The F1 score is the harmonic mean of precision and recall, providing a balanced measure of model performance (Iwendi et al., 2020; Dixit, 2022).


$$F1-\mathrm{score}=\frac{2*PR*\mathrm{Recall}}{PR+\mathrm{Recall}}$$

The study computed and compared these classification performance metrics for each of the seven ML algorithms: RF, SVM, CART, LR, GBM, kNN, and AdaBoot. The comparative performance analysis assessed the predictive performance degradation rates under varying distribution.

###### 3.1.4.4.4 Baseline model training and testing

In pursuit of achieving accurate FMD predictions in Uganda, this study commenced by undertaking baseline model training, testing and validation (Figure 9). The study began by segmenting the curated dataset from 2011 to 2020 (source) into two distinctive subsets using the train_test_split method in Python with random sampling: 70% allocated for training and 30% for testing. Random sampling ensures that each data point has an equal chance of being included in either the training or testing set, which helps to minimize bias and ensure that the resulting model’s performance is representative of its generalization ability (Mehrabi et al., 2021). Therefore, random sampling technique ensures that the training and testing sets are independent and identically distributed (i.i.d.), which is essential for evaluating the model’s performance accurately. The deliberate segregation of the source dataset was a critical step, ensuring a robust assessment of the predictive capabilities of the developed models. Each algorithm offered unique strengths and learning approaches, which aimed to leverage for the most effective predictive model.

The experimental approach encompassed a systematic procedure for each algorithm (Figure 9). The study initiated the training phase, utilizing 70% of source dataset. During this phase, the models analyzed the data, identifying intricate patterns and relationships crucial for accurate predictions. This intensive training phase was fundamental for each model to grasp the underlying features characterizing FMD outbreaks in Uganda. Subsequently, transitioned to the testing phase, employing the remaining 30% of the dataset. This independent subset played a vital role in rigorously evaluating each model’s predictive capabilities and ability to generalize when faced with previously unseen data with similar distribution. The predictive performance results from this phase would be vital in assessing the degradation rates for the various models under distribution shifts.

###### 3.1.4.4.5 Optimal performing model under stationary environment

The process of choosing the best-performing model among the experimented class imbalance handling techniques involved combining individual performance metric scores into a single measure, the weighted average performance core which was subsequently used for ranking the model performance. The process was guided by the following steps:

- *Assign weights:* Based on the relative importance for each performance metric, the study assigned an equal weight of 1 across all the metrics.
- *Calculate the weighted scores:* Multiplied each performance metric by its corresponding weight and summed up the results.
- *Compute weighted average scores:* Divided the sum of the weighted scores from (b) by the total number of performance metrics.

Therefore, the formula for calculating the weighted average score for n metrics is as follows:


$$\mathrm{Weighted}\;\mathrm{average}\;\mathrm{score}=\frac{\sum_{i=1}^{n}W_{i}\times M_{i}}{n}$$

*Where:*

$W_{i}$ represents the weight assigned to metric i , $M_{i}$ represents the value of metric, i and

n is the total number of metrics.

###### 3.1.4.4.6 Validating baseline model under distribution shifts

To validate the performances of the seven baseline models under distribution shifts in predicting FMD outbreaks, the study utilized the sequentially sampled target dataset (2021–2022). To quantify the impact of distribution shifts on these models, the study computed the *degradation rates* of the selected performance metrics, including accuracy, Area Under the Curve (AUC), recall, F1-score, and precision (Khattak et al., 2022). The degradation rates for each metric i of every model n were computed using the formula below.


$$\mathrm{Performance}\;\mathrm{Degradation}\;\mathrm{Rate}=\frac{\left(P_{\mathrm{test}}-P_{\mathrm{target}}\right)}{P_{\mathrm{test}}}\times 100\%$$

*Where:*

$P_{\mathrm{test}}$ represents the performance for matric iandcorrespondingmodeln ,

$P_{\mathrm{target}}$ represents the performance for matric iandcorrespondingmodeln .

This systematic approach allowed the study to gauge the reduction in performance metrics, serving as crucial indicators of the influence of distribution shifts on model efficacy. By quantifying the degradation rates across multiple performance metrics, the study comprehensively understood how the change in data distribution affected the models’ predictive abilities.

###### 3.1.4.4.7 Analyzing key predictive features for FMD outbreaks

The analysis of feature importance is a critical aspect within machine learning models, offering invaluable insights into the contribution and influence of individual features or variables on predictive outcomes (Kumar et al., 2020; Feng et al., 2021). Understanding the relative importance of these features aids in comprehending their impact on the model’s predictive power. To delve into feature importance, the study leveraged the feature_importances_ attribute, a model-specific attribute associated with the algorithm that exhibited superior performance in predicting FMD outbreaks in Uganda. The study generated feature importance values for the selected models by utilizing this attribute. This analysis holds immense significance as it reveals which variables are pivotal in predicting FMD outbreaks within the machine learning models. Identifying such influential factors is instrumental in refining models, enhancing predictive accuracy, and strategically allocating resources toward the most impactful variables. By scrutinizing the importance of features across various models, the researchers comprehensively understand the primary drivers behind the strategies for predicting FMD outbreaks.

## 4 Results

In this section, the study reveals the research findings related to creation of a unified and curated FMD dataset and assessment of performance degradation rates under varying distribution in Uganda. The comprehensive investigation unfolds in two significant sections: a unified and curated dataset, and assessment of predictive performance degradation rates under varying distribution. Each section sheds light on distinct yet interconnected aspects.

### 4.1 A unified and curated FMD dataset

Through comprehensive data pre-processing, the study addressed missing values and outlier data points, resulting in the creation of a unified and curated FMD dataset. This pre-processed dataset is essential for constructing ML-based predictive models for FMD outbreaks. By ensuring the data’s accuracy and consistency, the study enhances the reliability and effectiveness of ML-based models, which are critical for early detection and optimal allocation of resources to mitigate FMD outbreaks.

#### 4.1.1 Data collection and sources

*Historical FMD Data:* Data was collected from NADDEC and WOAH, covering a period of 12 years from 2011 to 2022. This data included 12,484 records detailing the time and location of outbreaks, confirmed cases, animals at risk, and animal density as indicated in Table 4.

**Table 4:** Raw FMD dataset.

Features	Description	Source	Data type	Total records	Missing values	Duplicates	Outliers
rainfall	Rainfall	UNMA	Continuous	12,484	37 (0.3%)	100 (0.8%)	12 (0.1%)
max_temp	Maximum temperature	UNMA	Continuous				
cattle_density	Animal density	NADDEC	Continuous				
adjacent_national_parks	Adjacent to national parks	Pennsylvania State University	Categorical				
adjacent_international_border	Adjacent to international border	Pennsylvania State University	Categorical				

*Risk Factor Data:* Rainfall and maximum temperature data were obtained from UNMA, while proximity to protected areas and international borders was sourced from the Pennsylvania State University.

#### 4.1.2 Data pre-processing

*Data Cleaning:* Initial data contained 0.3% missing values, 0.8% duplicates and 0.1% outliers. Missing and outlier values were handled using mean imputation, and duplicates were removed, resulting in a clean dataset with complete records.

*Data Integration:* Datasets were merged using common primary keys including location and time. Temporal data was aligned to ensure consistency across all records.

#### 4.1.3 Final unified and curated dataset composition

The unified and curated dataset comprised a total of 12,384 records from 86 districts. Of these records, 97.88% represented non-outbreaks, while only 2.12% represented outbreaks, highlighting a significant class imbalance in the FMD dataset, as shown in Supplementary Table 1. This imbalance is crucial to consider as it can impact the performance of machine learning models trained on this data. Additionally, the prevalence of FMD outbreaks across different districts varies significantly, as illustrated in Supplementary Figure 5.

### 4.2 Assessing baseline predictive performance degradation rates in a non-stationary environment

To assess the impact of distribution shifts on the predictive performance of machine learning-based models for FMD outbreaks in Uganda, the study employed a comprehensive experimental methodology. Seven classification machine learning algorithms were carefully selected, trained, tested, and validated. To present the results effectively, the study adopted a structured three-phase approach: Phase 1 (Section 4.2.1.1) involved presenting the test results for the baseline models with imbalanced dataset. Phase 2 (Section 4.2.1.2) focused on presenting the test results for the baseline models using a randomly under-sampled dataset, aiming to address the class imbalance issue. Phase 3 (Section 4.2.1.3) encompassed presenting the test results for the baseline models utilizing various over-sampling techniques, including SMOTE (original), Borderline-SMOTE, SMOTE-SVM, and ADASYN, to further explore the impact of balancing techniques on model performance. From Phase 1 to 3, the study tested the baseline models under stationary environment. Finally, Phase 4 (Section 4.2.2.1) presents the results regarding the model performance degradation rates under distribution shifts, shedding light on the vulnerability of ML-based models to changes in data distribution.

#### 4.2.1 Baseline model test performance under stationary environment

To comprehend the influence of distribution shifts on the predictive accuracy of ML-based models for FMD outbreaks, the study initially assessed the performance of baseline models in a stationary environment (section 4.2.1). Subsequently, in section 4.2.2, it examined performance under distribution shifts and conducts a comparative analysis for the performance degradation effect.

##### 4.2.1.1 Phase 1: model performance with imbalanced classes

Examining the baseline models that were trained and tested on imbalanced dataset, Supplementary Table 2 reveals notably poor performance across all metrics, with bold values depicting the highest performance. This subpar predictive capability primarily stems from the substantial class imbalance present within the FMD dataset. The imbalance in class distribution poses a significant challenge for the ML-based models to accurately predict occurrences of FMD outbreaks, leading to lower performance across various evaluation metrics.

##### 4.2.1.2 Phase 2: model performance with randomly undersampled dataset

Under-sampling the majority class (non-outbreak) to balance it with the minority class (outbreak) resulted in only marginal performance improvement, with the overall performance remaining poor across all metrics. The best performance is depicted in bold values, as highlighted in Supplementary Table 3. The poor performance can be attributed to the limited dataset used for training the baseline models.

##### 4.2.1.3 Phase 3: baseline model performance with oversampled dataset

**Figure 10:**
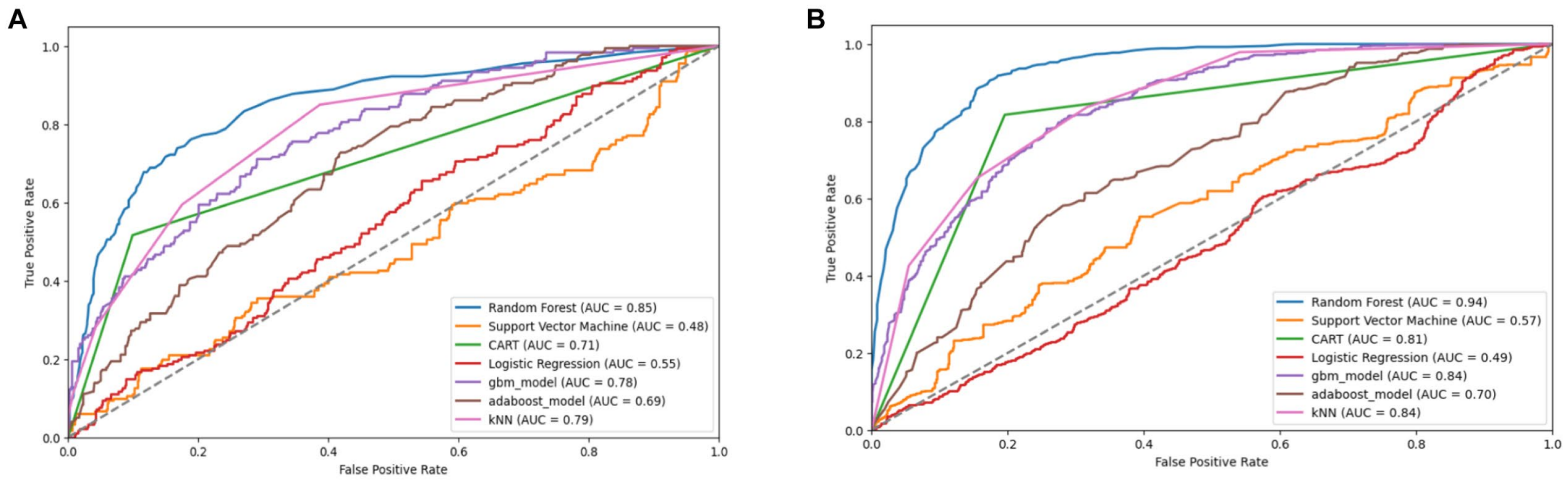
Combined AUC-ROC performance of baseline models with minority class oversampled by a factor of 20 **(A)** and balanced dataset **(B)** using SMOTE (original).

**Figure 11 fig11:**
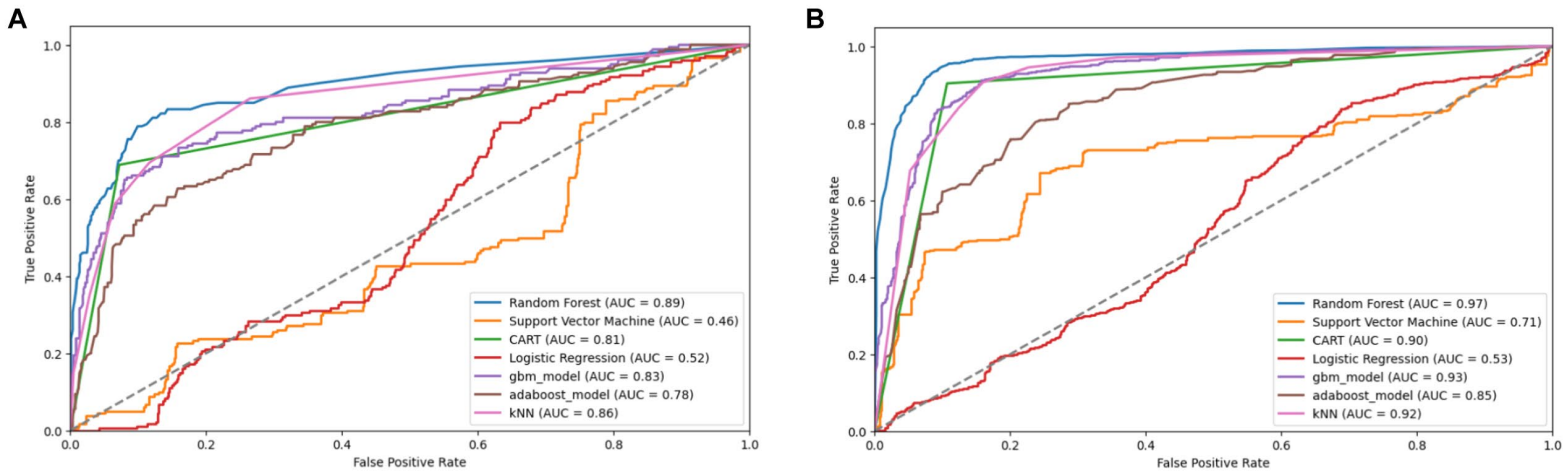
Combined AUC-ROC performance of baseline models with minority class oversampled by a factor of 20 **(A)** and balanced dataset **(B)** using borderline SMOTE.

**Figure 12 fig12:**
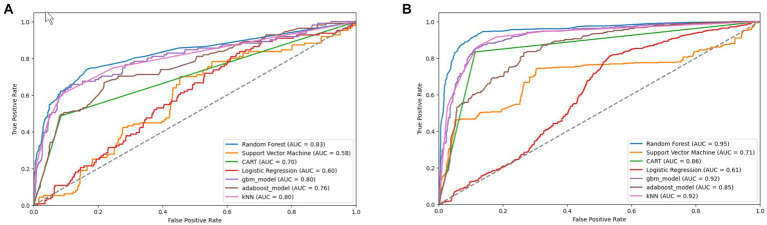
Combined AUC-ROC performance of baseline models with minority class oversampled by a factor of 20 **(A)** and balanced dataset **(B)** using SMOTE-SVM.

**Figure 13:**
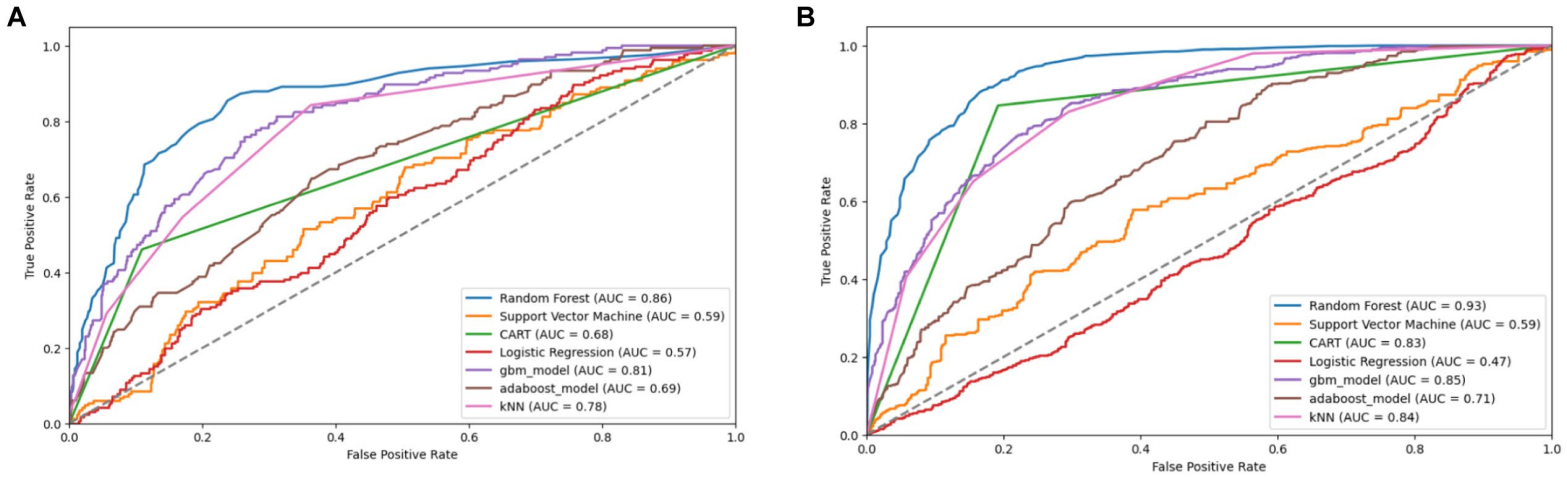
Combined AUC-ROC performance of baseline models with minority class oversampled by a factor of 20 **(A)** and balanced dataset **(B)** using ADASYN.

The original SMOTE algorithm and its three variants, including Borderline-SMOTE, SMOTE-SVM, and ADASYN, were explored to address the imbalanced dataset and enhance the baseline model performances for predicting FMD outbreaks in Uganda. The study compares the findings between two scenarios: one where the minority samples were oversampled by a factor of 20 and the other where the minority samples were oversampled to achieve balance with the majority class. Results from the oversampling process indicate that baseline models trained on a balanced dataset for all techniques consistently outperformed those trained on minority samples oversampled by a factor of 20. The highest performance is depicted in bold values, as shown in Tables 5 [Table tab6] [Table tab7]–8. Similarly, Figures 10 [Fig fig11] [Fig fig12]–13 visualize the performance across the SMOTE and itsvariants.

**Table 5:** Comparative analysis of baseline model performance with minority class oversampled by a factor of 20 and balanced dataset using SMOTE (original).

SMOTE (original) test results										
Dataset before oversampling: no-outbreak – 2769; outbreak – 111										
Dataset after oversampling: no-outbreak – 2769; outbreak – 2220 (aug. by 20)						Dataset after oversampling: no-outbreak – 2769; outbreak – 2769 (Balanced)				
Training dataset: 70%						Training dataset: 70%				
Test dataset: 30%						Test dataset: 30%				
Model	ACC	AUC	Recall	Precision	F1-score	ACC	AUC	Recall	Precision	F1-score
RF	#	0.65	0.42	0.70	**0.53**	**0.86**	**0.94**	**0.90**	**0.83**	**0.86**
SVM	#	0.48	0.00	0.00	0.00	0.57	0.57	0.56	0.56	0.56
GBM	#	0.78	0.06	0.56	0.11	0.75	0.84	0.82	0.70	0.76
CART	#	0.71	**0.52**	0.53	**0.53**	0.81	0.81	0.82	0.80	0.81
LR	#	0.55	0.00	0.00	0.00	0.51	0.49	0.62	0.49	0.55
kNN	#	**0.79**	0.28	0.59	0.38	0.76	0.84	0.84	0.71	0.77
AdaBoost	#	0.69	0.02	**0.80**	0.04	0.63	0.70	0.66	0.61	0.63

**Table 6 tab6:** Comparative analysis of baseline model performance with minority class oversampled by a factor of 20 and balanced dataset using borderline SMOTE.

Borderline SMOTE Test Results										
Dataset before Oversampling: no-outbreak – 2769; outbreak – 111										
Dataset after Oversampling: no-outbreak – 2769; outbreak – 2220 (aug. by 20)						Dataset after Oversampling: no-outbreak – 2769; outbreak – 2769 (Balanced)				
Training dataset: 70%						Training dataset: 70%				
Test dataset: 30%						Test dataset: 30%				
Model	ACC	AUC	Recall	Precision	F1-score	ACC	AUC	Recall	Precision	F1-score
RF	#	**0.89**	0.58	**0.77**	0.66	**0.92**	**0.97**	**0.94**	**0.90**	**0.92**
SVM	#	0.46	0.00	0.00	0.00	0.70	0.71	0.73	0.68	0.70
GBM	#	0.83	0.46	0.74	0.57	0.87	0.93	0.86	0.86	0.86
CART	#	0.81	**0.69**	0.68	**0.68**	0.90	0.90	0.90	0.89	0.90
LR	#	0.52	0.00	0.00	0.00	0.52	0.53	0.05	0.52	0.10
kNN	#	0.86	0.59	0.66	0.62	0.87	0.92	0.91	0.84	0.87
AdaBoost	#	0.78	0.21	0.60	0.31	0.77	0.85	0.81	0.74	0.77

**Table 7 tab7:** Comparative analysis of baseline model performance with minority class oversampled by a factor of 20 and balanced dataset using SMOTE-SVM.

SMOTE-SVM Test Results										
Dataset before Oversampling: no-outbreak – 2769; outbreak – 111										
Dataset after Oversampling: no-outbreak – 2769; outbreak – 2220 (aug. by 20)						Dataset after Oversampling: no-outbreak – 2769; outbreak – 2769 (Balanced)				
Training dataset: 70%						Training dataset: 70%				
Test dataset: 30%						Test dataset: 30%				
Model	ACC	AUC	Recall	Precision	F1-score	ACC	AUC	Recall	Precision	F1-score
RF	#	**0.83**	0.45	0.66	**0.53**	**0.91**	**0.95**	**0.88**	**0.86**	**0.87**
SVM	#	0.58	0.00	0.00	0.00	0.70	0.71	0.68	0.57	0.62
GBM	#	0.80	0.12	**0.72**	0.20	0.87	0.92	0.81	0.83	0.82
CART	#	0.70	**0.49**	0.44	0.46	0.87	0.86	0.83	0.80	0.82
LR	#	0.60	0.00	0.00	0.00	0.65	0.61	0.00	0.00	0.00
kNN	#	0.80	0.46	0.58	0.51	0.87	0.92	0.86	0.80	0.83
AdaBoost	#	0.76	0.03	0.60	0.05	0.79	0.85	0.61	0.73	0.67

**Table 8:** Comparative analysis of baseline model performance with minority class oversampled by a factor of 20 and balanced dataset using ADASYN.

ADASYN test results										
Dataset before Oversampling: no-outbreak – 2769; outbreak – 111										
Dataset after Oversampling: no-outbreak – 2769; outbreak – 2220 (aug. by 20)						Dataset after Oversampling: no-outbreak – 2769; outbreak – 2769 (Balanced)				
Training dataset: 70%						Training dataset: 70%				
Test dataset: 30%						Test dataset: 30%				
Model	ACC	AUC	Recall	Precision	F1-score	ACC	AUC	Recall	Precision	F1-score
RF	#	**0.86**	0.34	0.64	0.44	**0.85**	**0.93**	**0.90**	**0.81**	**0.85**
SVM	#	0.59	0.00	0.00	0.00	0.58	0.59	0.61	0.55	0.58
GBM	#	0.81	0.08	**0.88**	0.15	0.76	0.85	0.87	0.70	0.78
CART	#	0.68	**0.46**	0.46	**0.46**	0.83	0.83	0.85	0.80	0.82
LR	#	0.57	0.00	0.00	0.00	0.49	0.47	0.56	0.47	0.51
kNN	#	0.78	0.29	0.51	0.37	0.76	0.84	0.83	0.72	0.77
AdaBoost	#	0.69	0.04	0.75	0.07	0.64	0.71	0.70	0.61	0.65

When considering the oversampled balanced dataset across all techniques, the Random Forest (RF) model consistently demonstrated the most impressive performance among the seven machine learning algorithms utilized in the study. Across all techniques where the classes were balanced, RF achieved an accuracy of 85% and above, indicating its high precision in making correct predictions. Moreover, RF showcased an AUC value of 0.93 and above, implying a strong ability to distinguish between positive and negative cases and offering excellent overall model performance. Additionally, RF attained high values for precision (0.81) and above, recall (0.88) and above, and F1 score (0.85) and above, signifying its balanced performance across various evaluation criteria (Supplementary Table 3 and Tables 5–7).

##### 4.2.1.4 Optimal baseline model performance under stationary environment

**Figure 14:**
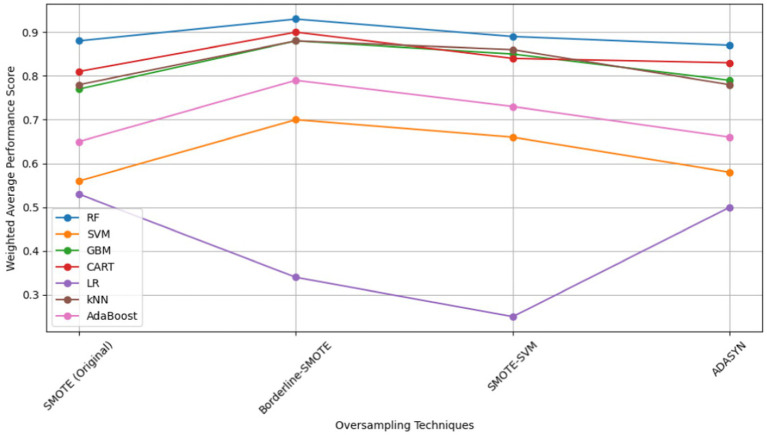
Visual overview of baseline model performance across oversampling techniques.

To determine the most effective baseline model among the experimented oversampling techniques, the study calculated the weighted average scores, which were utilized to rank their performance, as illustrated in Table 9, with bold values showing the highest performance. Among all the experimented models, RF emerged as the best-performing model across the board, as depicted in Figure 14. Similarly, Borderline-SMOTE technique demonstrated superiority as the most effective oversampling technique for mitigating class imbalance and improving the prediction of FMD outbreaks in Uganda, as evidenced in Figure 14.

**Table 9:** Weighted average performance scores of baseline models across oversampling techniques.

Weighted average performance scores				
Model	SMOTE (original)	Borderline-SMOTE	SMOTE-SVM	ADASYN
RF	**0.88**	**0.93**	**0.89**	**0.87**
SVM	0.56	0.70	0.66	0.58
GBM	0.77	0.88	0.85	0.79
CART	0.81	0.90	0.84	0.83
LR	0.53	0.34	0.25	0.50
kNN	0.78	0.88	0.86	0.78
AdaBoost	0.65	0.79	0.73	0.66

#### 4.2.2 Baseline model validation performance under distribution shifts

In this section, the study presents the validation performance of the baseline models under varying distributions. It includes a comparative analysis of baseline model performance, highlighting the rates of performance degradation.

##### 4.2.2.1 Phase 4: baseline model performance

Based on the results presented in Table 9, the Borderline SMOTE technique emerges as the most effective method for addressing class imbalance within the FMD dataset. Therefore, the baseline model test performances obtained under the Borderline SMOTE technique are considered as the reference results for evaluating the impact of distribution shifts on the predictive capability of the seven selected machine learning models for predicting FMD outbreaks in Uganda. In this section, the study presents findings that illustrate the influence of distribution shifts on the predictive performance of the baseline models, as depicted in Supplementary Table 4 and Table 10, with bold values showing the highest performance. The results indicate significant degradation rates across all models.

**Table 10:** Comparative model performance between in-distribution and out-of-distribution settings.

Dataset after Borderline SMOTE Oversampling: no-outbreak – 2769; outbreak – 2769 (Balanced)						Validation Dataset after Borderline SMOTE Oversampling: no-outbreak – 554; outbreak – 554 (Balanced)					Model Performance Degradation Rates (%)				
Training dataset: 70%; Test dataset: 30%															
In-Distribution Performance						Out-of-Distribution Performance									
Model	ACC	AUC	Recall	Precision	F1-score	ACC	AUC	Recall	Precision	F1-score	ACC	AUC	Recall	Precision	F1-score
RF	**0.92**	**0.97**	**0.94**	**0.90**	**0.92**	0.46	0.58	0.03	0.24	0.06	**50.00**	40.21	96.81	73.33	**93.48**
SVM	0.70	0.71	0.73	0.68	0.70	0.45	0.56	0.23	0.41	0.30	35.71	21.13	68.49	39.71	57.14
GBM	0.87	0.93	0.86	0.86	0.86	**0.53**	0.48	0.23	0.56	0.32	39.08	48.39	73.26	34.88	62.79
CART	0.90	0.90	0.90	0.89	0.90	0.49	0.49	0.11	0.44	0.17	45.56	45.56	87.78	50.56	81.11
LR	0.52	0.53	0.05	0.52	0.10	0.48	**0.65**	0.00	0.08	0.01	7.69	22.64	**100.00**	**84.62**	90.00
kNN	0.87	0.92	0.91	0.84	0.87	0.53	0.52	**0.26**	**0.56**	**0.35**	39.08	43.48	71.43	33.33	59.77
AdaBoost	0.77	0.85	0.81	0.74	0.77	0.45	0.41	0.18	0.39	0.25	41.56	**51.76**	77.78	47.30	67.53

###### 4.2.2.1.1 Baseline model performance degradation rates

When assessing the performance of the seven baseline models on the target (validation) dataset, the study noted varying degrees of performance, highlighting the impact of distribution shifts on model performances in predicting FMD outbreaks (Table 10). Each model displayed distinct characteristics concerning accuracy, sensitivity, precision, and specificity under these conditions.

Random Forest (RF), initially displaying superior overall performance in the absence of distribution shifts, saw a significant decrease in accuracy (ACC) by 50% and a notable decline of 40.21% in the Area Under the Curve (AUC) value of the Receiver Operating Characteristic (ROC) curve. Additionally, RF experienced reductions in Recall by 96.81%, Precision by 73.33%, and F1-score by 93.48%. Support Vector Machine (SVM) encountered reductions in accuracy (ACC) by 35.71%, AUC by 21.13%, Recall by 68.49%, Precision by 39.71%, and F1-score by 57.14%. Gradient Boosting Machine (GBM) saw decreases in accuracy (ACC) by 39.08%, AUC by 48.39%, Recall by 73.26%, Precision by 34.88%, and F1-score by 62.79%. Classification and Regression Trees (CART) experienced declines in accuracy (ACC) by 45.56%, AUC by 45.56%, Recall by 87.78%, Precision by 50.56%, and F1-score by 81.11%. Logistic Regression (LR) encountered reductions in accuracy (ACC) by 7.69%, Recall by 100.00%, Precision by 84.62%, and F1-score by 90.00%, yet LR demonstrated improved performance for AUC by 22.64%, attributed to its incorporation of regularization techniques including L1 and L2. k-Nearest Neighbors (kNN) experienced reductions in accuracy (ACC) by 39.08%, AUC by 43.48%, Recall by 71.43%, Precision by 33.33%, and F1-score by 59.77%. AdaBoost saw decreases in accuracy (ACC) by 41.56%, AUC by 51.76%, Recall by 77.78%, Precision by 47.30%, and F1-score by 67.53%. These findings underscore the considerable influence of distribution shifts on the predictive performance of ML-based algorithms across various evaluation metrics.

###### 4.2.2.1.2 Feature importance

**Figure 15:**
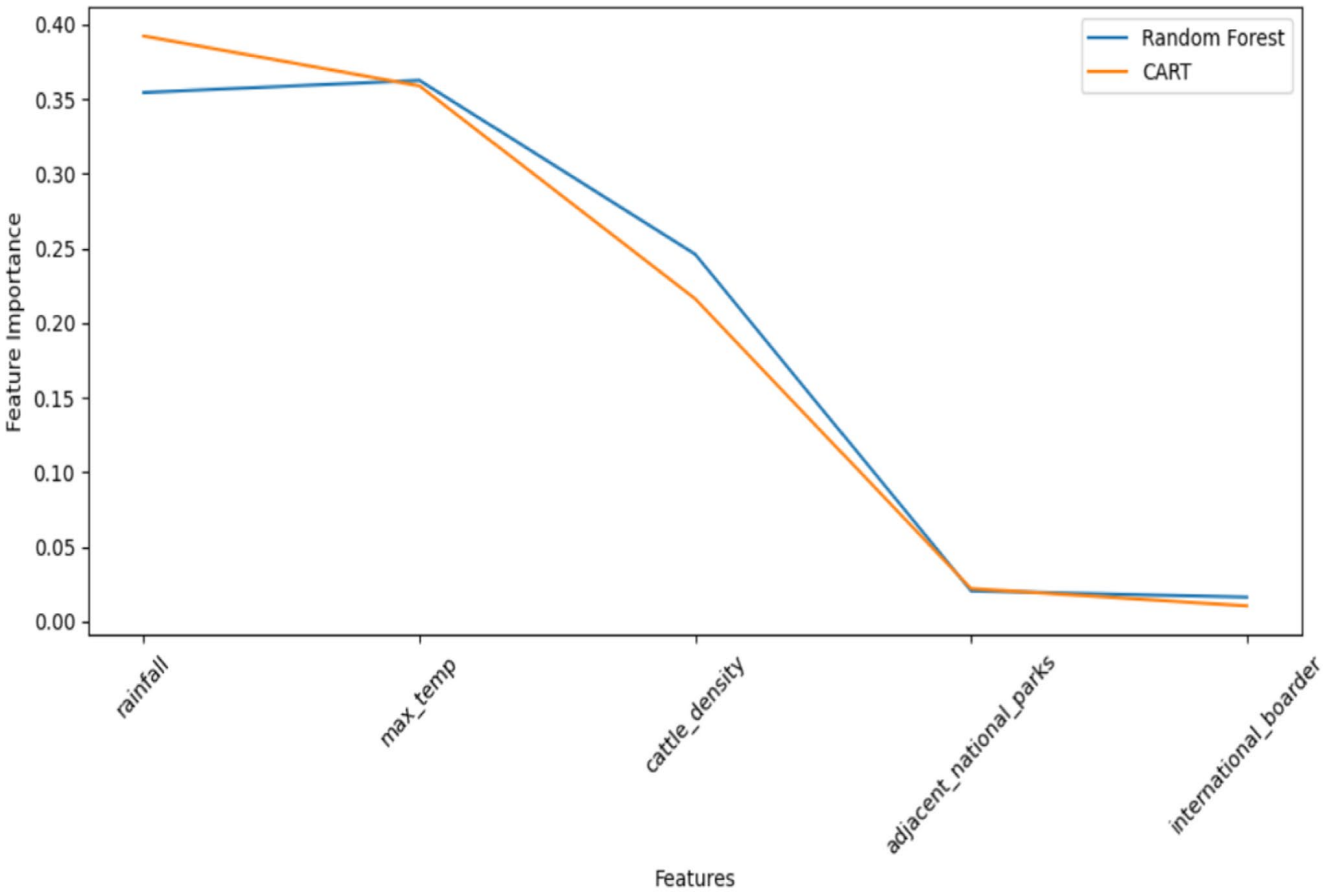
Comparison of feature importance for RF and CART models.

In predicting FMD outbreaks in Uganda, the importance of features played a pivotal role in enhancing the understanding and predictive capabilities of the models. Feature importance refers to the measure of how much each input feature contributes to the predictive power of a machine learning model (Kumar et al., 2020; Feng et al., 2021). It provides insights into which features have the most significant impact on the model’s performance and can help identify the key factors influencing the occurrence of FMD outbreaks. The study used the Random Forest (RF) model, which demonstrated superior predictive performance, and CART to assess feature importance (Figure 15). Based on the importance results, the following features were found to have the most significant impact on FMD outbreak prediction:

- Rainfall: This feature exhibited the highest level of importance, signifying its strong association with FMD outbreaks. Low rainfall may create conditions conducive to the disease’s transmission and, as such, serves as an essential early warning indicator.
- Max temperature: Max temperature was identified as the second most important feature. Temperature can influence disease vectors, animal behavior, and the survival of the virus, making it crucial in predicting outbreaks.
- Cattle density: The density of cattle populations was the third most important feature. High cattle density areas may experience a more rapid spread of FMD, making this a critical factor to consider in preventive measures.
- Proximity to adjacent parks: The proximity of areas to protected wildlife zones was identified as the fourth most important feature. These regions may serve as reservoirs for the disease, increasing the risk of outbreaks in nearby livestock populations.
- Proximity to international borders: Closeness to international borders rounded out the list of important features. Border areas may be more susceptible to the introduction of the virus through cross-border movements of animals.

By recognizing the importance of these features, the study emphasizes the need to focus on these variables when implementing preventive strategies and early warning systems. It is clear that understanding the importance of features significantly contributes to developing effective measures for managing and controlling FMD outbreaks in Uganda.

As indicated, rainfall and maximum temperature contribute significantly to the predictive power of the models, followed by cattle density. Proximity to national parks and the international border contributes little to the predictive power.

## 5 Discussion of results

The study aimed, firstly, to create a unified and curated dataset for Foot-and-Mouth Disease (FMD) in Uganda. This was achieved by utilizing a retrospective approach to collect disparate datasets from various sources and conducting experiments to address missing data and outliers. Secondly, the study aimed to assess the performance degradation rates under varying distribution. This was accomplished by training machine learning models on the unified and curated FMD dataset, testing them, and evaluating their predictive performance on the holdout dataset to measure the impact of variability in the dataset. This section presents a discussion of the study’s findings, contributions, limitations, and recommendations.

### 5.1 A unified and curated dataset for FMD

The study retrospectively collected historical data on FMD outbreaks and the factors influencing their occurrences, disparate datasets were pre-processed to create a unified and curated dataset for FMD in Uganda. The statistical results provide significant evidence of class imbalance, which is known to impact performance in the ML domain. Predictions tend to be biased toward the majority class, as the number of FMD non-outbreaks were significantly greater than the number of outbreaks.

### 5.2 ML-based predictive performance degradation under varying distribution

The study investigated seven ML algorithms as baseline models for FMD outbreak prediction. Notably, significant impacts of class imbalance on the predictive performance of these algorithms were observed when using the randomly sampled test dataset. The poor performance was observed across multiple evaluation metrics, including area under the curve (AUC), recall, precision, and F1-score. Such consistent poor performance highlighted the critical need for addressing the class imbalance problem for improved performance in prediction of FMD outbreaks in Uganda. To mitigate class imbalance in the FMD dataset, various data augmentation techniques were explored, including random undersampling, SMOTE (Original), Borderline-SMOTE, SMOTE-SVM, and ADASYN.

The findings revealed that oversampling techniques led to substantial improvements in model performance, particularly when the classes were balanced. Among these techniques, Borderline-SMOTE emerged as the most effective, attributed to its superior handling of noise through synthetic sample generation. Additionally, among the seven models examined, random forest (RF) exhibited superior performance across all evaluation metrics including accuracy, AUC, recall, precision and F1-score on the test dataset. This can be attributed to its ensemble nature, where it combines various decision trees to enhance predictive accuracy. However, when validated with a target dataset exhibiting varying distributions, all models experienced significant degradation across all performance metrics. These findings underscore the significance of addressing distribution shifts in FMD outbreak prediction.

### 5.3 Limitations of the study

This section acknowledges the limitations encountered during the study and discusses their potential impact on the results:

- While this study focused on five key risk factors including rainfall, temperature, proximity to international borders, proximity to national parks, and cattle density as predictors for FMD outbreaks, it acknowledges the potential importance of other factors. These include animal movement, animal trade, water sources, and breeding methods, which could further enhance the predictive performance of machine learning models.
- The study was conducted within the endemic settings of Uganda. Consequently, the predictors identified may be unique to Uganda’s context, impacting the generalizability of the findings to other regions.
- Variations in FMD outbreak reporting practices can lead to inconsistencies in the data. Some regions have better reporting mechanisms for FMD outbreaks, while others may underreport or overreport cases.
- The use of performance degradation rates across metrics to detect distribution shifts in the FMD dataset is prone to trigger false alarms, prompting retraining which is time-consuming and costly.

## 6 Conclusion

This study aimed to explore the predictive capabilities of machine learning models for Foot and Mouth Disease outbreaks in Uganda by creating a unified dataset and evaluating model performance under varying distribution conditions. The unified dataset highlighted significant class imbalances in FMD outbreak data, a critical challenge for accurate predictive modeling. Various data augmentation techniques, including SMOTE, borderline-SMOTE, SMOTE-SVM, and ADASYN, were explored to mitigate these imbalances. In a stationary environment, where data distributions were consistent, models such as Random Forest (RF) with borderline-SMOTE excelled on the test dataset, showcasing robust predictive performance. However, when validated under scenarios of varying distributions, all models exhibited notable performance degradation. This highlighted a critical limitation: the current models are not sufficiently robust to reliably predict FMD outbreaks in Uganda when environmental conditions change. The findings underscore the need for future research to focus on advancing both data-centric and model-centric approaches. Specifically, efforts should explore advanced techniques in domain adaptation to effectively handle the challenges posed by varying distributions in FMD outbreak prediction. Furthermore, integrating additional predictors such as animal movement patterns, trade data, and ecological factors could enhance the predictive power of models. These enhancements are crucial for improving preparedness and response strategies against FMD outbreaks, not only in Uganda but also in other endemic regions globally.

## Data availability statement

Publicly available datasets were analyzed in this study. This data can be found at: http://fleet.naro.go.ug/mileyplc/FMD_outbreaks_dataset_risk_current_1.csv; http://fleet.naro.go.ug/mileyplc/FMD_outbreaks_dataset_risk_reference_1.csv.

## Author contributions

GK: Conceptualization, Data curation, Formal analysis, Funding acquisition, Investigation, Methodology, Project administration, Resources, Software, Supervision, Validation, Visualization, Writing – original draft, Writing – review & editing. FK: Writing – original draft, Writing – review & editing. SK: Writing – original draft, Writing – review & editing. DJ: Writing – original draft, Writing – review & editing. SB: Writing – original draft, Writing – review & editing. JR: Writing – original draft, Writing – review & editing. PS: Writing – original draft, Writing – review & editing. SM: Writing – original draft, Writing – review & editing. YK: Writing – original draft, Writing – review & editing.
