# Supplementary material for: A unified Foot and Mouth Disease dataset for Uganda: evaluating machine learning predictive performance degradation under varying distributions
Source: Front Artif Intell. 2024 Jul 31;7:1446368. doi: 10.3389/frai.2024.1446368 (PMC11322090; doi:10.3389/frai.2024.1446368)

**Supplementary Material**

Supplementary Table 1: Statistical Characteristics of the Unified and Curated FMD Dataset

| **Total Records** | **Number of Features** | **Non-outbreaks** | **Outbreaks** |
| --- | --- | --- | --- |
| 12,384 | 5 | 12,122  (97.88%) | 262  (2.12%) |

Supplementary Table 2: Baseline Model Performance with Imbalanced Classes

| **Baseline Test Results** | | | | | |
| --- | --- | --- | --- | --- | --- |
| Original dataset: no-outbreak - 2769; outbreak - 111 | | | | | |
| Training dataset: 70% | | | | | |
| Test dataset: 30% | | | | | |
| **Model** | **ACC** | **AUC** | **Recall** | **Precision** | **F1-score** |
| RF | 0.97 | 0.55 | 0.00 | 0.00 | 0.00 |
| SVM | 0.97 | **0.58** | 0.00 | 0.00 | 0.00 |
| GBM | 0.97 | 0.57 | 0.00 | 0.00 | 0.00 |
| CART | 0.92 | 0.49 | **0.04** | **0.03** | **0.03** |
| LR | 0.97 | 0.51 | 0.00 | 0.00 | 0.00 |
| kNN | 0.97 | 0.52 | 0.00 | 0.00 | 0.00 |
| AdaBoost | 0.97 | 0.57 | 0.00 | 0.00 | 0.00 |

Supplementary Table 3: Baseline Model Performance with Undersampled Majority Class

| **Random Under-sampled Test Results** | | | | | |
| --- | --- | --- | --- | --- | --- |
| Dataset before Under-sampling: no-outbreak - 2769; outbreak - 111 | | | | | |
| Dataset after Under-sampling: no-outbreak - 111; outbreak - 111 | | | | | |
| Training dataset: 70% | | | | | |
| Test dataset: 30% | | | | | |
| **Model** | **ACC** | **AUC** | **Recall** | **Precision** | **F1-score** |
| RF | ### | 0.60 | **0.06** | 0.20 | 0.09 |
| SVM | ### | 0.40 | 0.00 | 0.00 | 0.00 |
| GBM | ### | 0.58 | **0.06** | 0.22 | 0.09 |
| CART | ### | 0.51 | 0.21 | 0.19 | **0.20** |
| LR | ### | 0.43 | 0.00 | 0.00 | 0.00 |
| kNN | ### | **0.63** | **0.06** | **0.25** | 0.10 |
| AdaBoost | ### | 0.45 | 0.03 | 0.17 | 0.05 |

Supplementary Table 4: Performance of Baseline Models under Distribution Shifts

| **Validation Results with Borderline SMOTE** | | | | | |
| --- | --- | --- | --- | --- | --- |
| Validation dataset: no-outbreak - 554; outbreak - 554 | | | | | |
|  |  |  |  |  |  |
| **Model** | **ACC** | **AUC** | **Recall** | **Precision** | **F1-score** |
| RF | 0.46 | 0.58 | 0.03 | 0.24 | 0.06 |
| SVM | 0.45 | 0.56 | 0.23 | 0.41 | 0.30 |
| GBM | **0.53** | 0.48 | 0.23 | **0.56** | 0.32 |
| CART | 0.49 | 0.49 | 0.11 | 0.44 | 0.17 |
| LR | 0.48 | **0.65** | 0.00 | 0.08 | 0.01 |
| kNN | 0.53 | 0.52 | **0.26** | **0.56** | **0.35** |
| AdaBoost | 0.45 | 0.41 | 0.18 | 0.39 | 0.25 |

Supplementary Figure 1: Prevalence of FMD Outbreaks across Districts
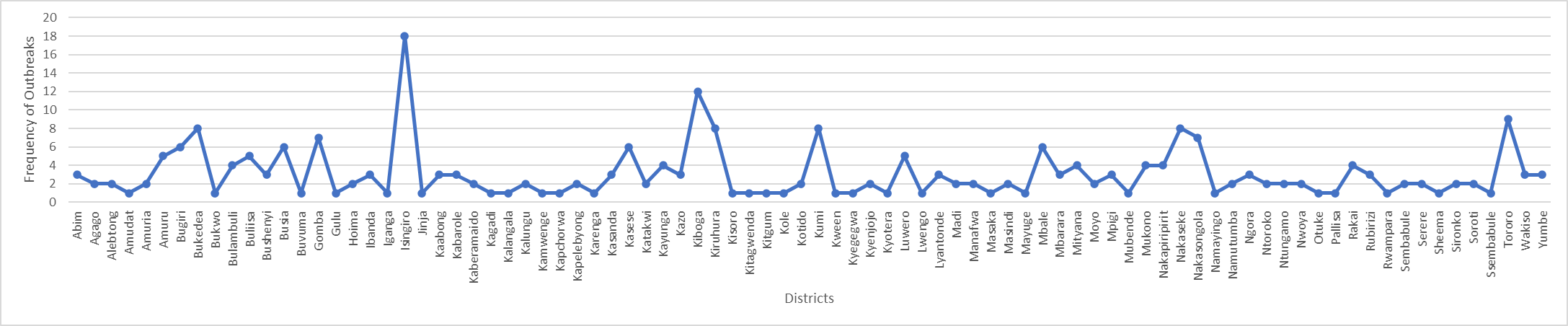


Supplementary Figure 2: Distribution of Imbalanced Dataset with Minority Samples by Months (A) and years (B)

| **A**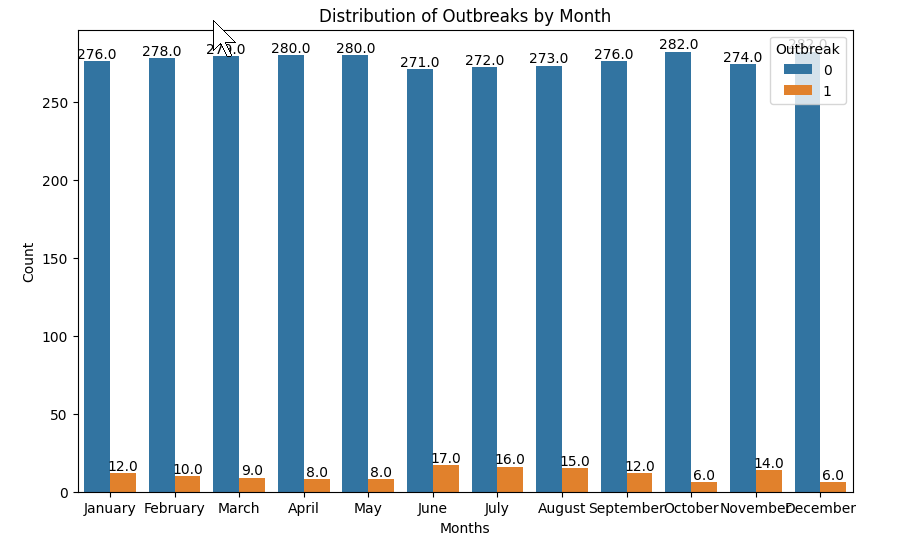 | **B**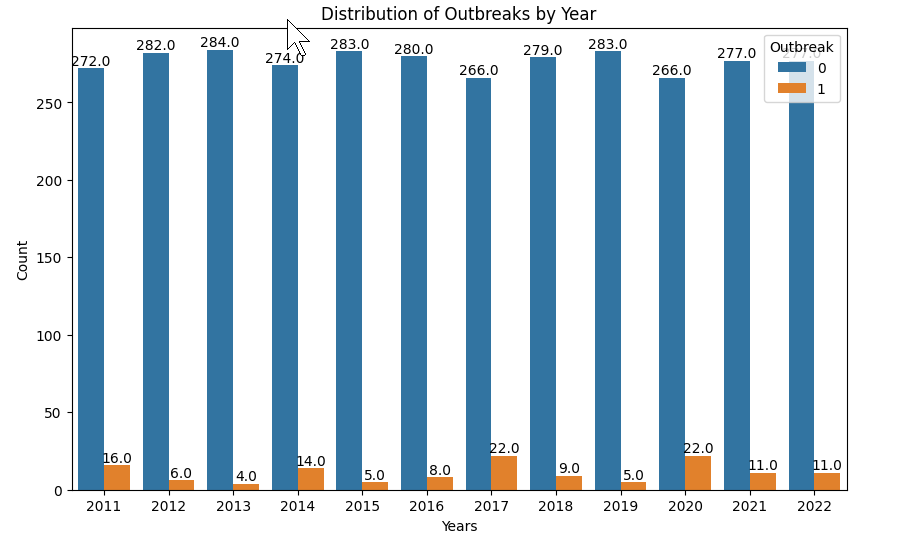 |
| --- | --- |

Supplementary Figure 3: Random Under-sampling the Majority Class for a Balanced Dataset


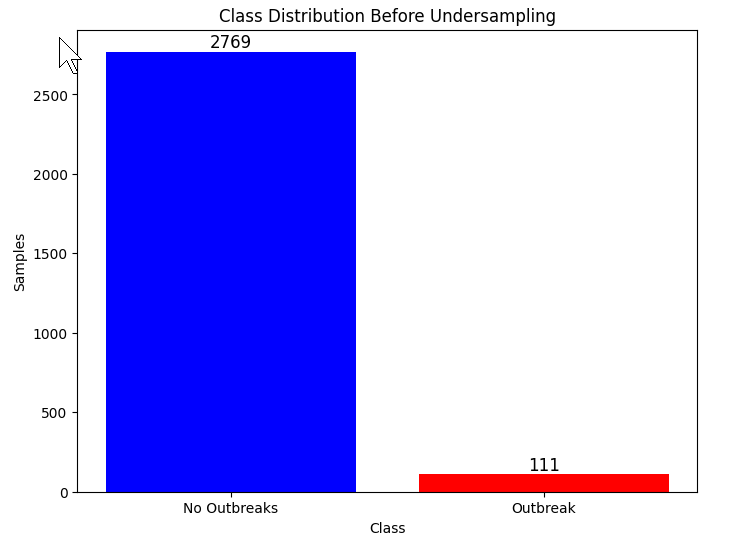

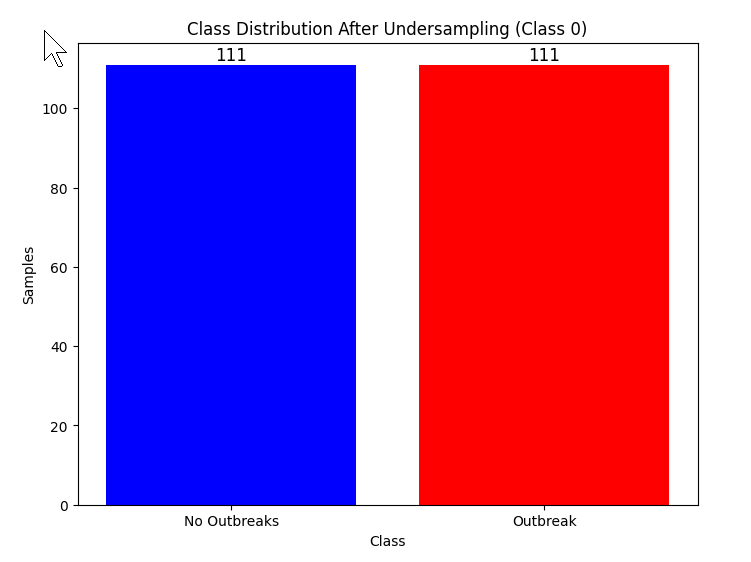


Supplementary Figure 4: Utilizing SMOTE (original), SMOTE-SVM, Borderline-SMOTE and ADASYN Techniques for oversampling the Minority Class with a Factor of 20


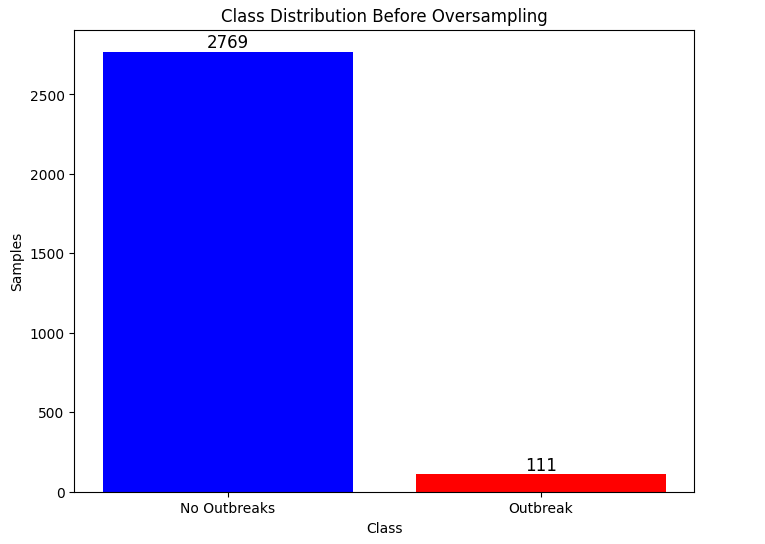

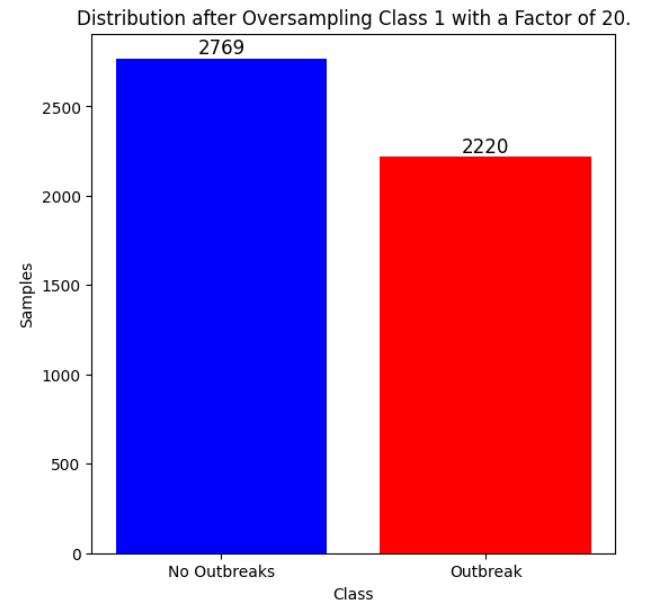


Supplementary Figure 5: Prevalence of FMD Outbreaks across Districts


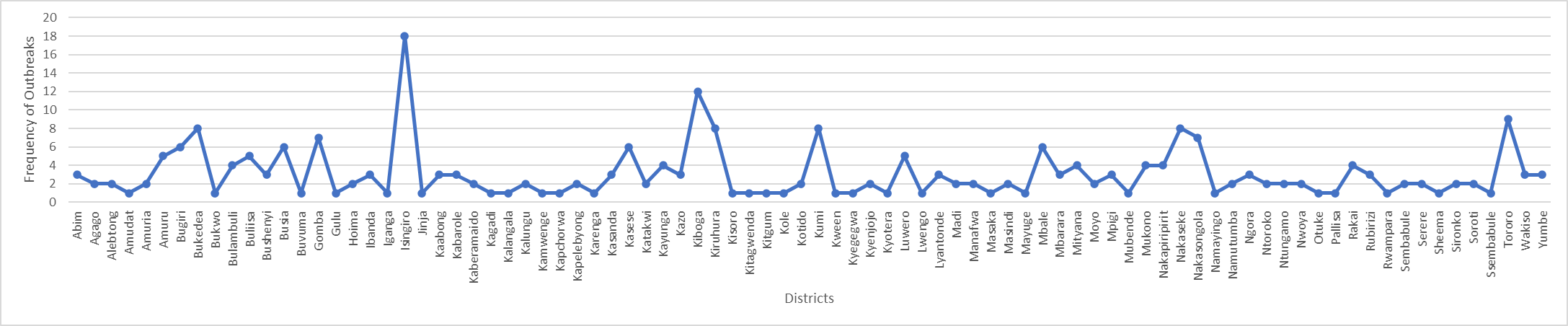

Supplement: Supplementary file 1 [file Data_Sheet_1.docx]
